# Integrated watershed management solutions for healthy coastal ecosystems and people

**DOI:** 10.1017/cft.2023.15

**Published:** 2023-05-05

**Authors:** Ama Wakwella, Amelia Wenger, Aaron Jenkins, Joleah Lamb, Caitlin D. Kuempel, Danielle Claar, Chris Corbin, Kim Falinski, Antonella Rivera, Hedley S. Grantham, Stacy D. Jupiter

**Affiliations:** 1 School of Earth and Environmental Sciences, University of Queensland, St Lucia, QLD, Australia; 2 Centre for Biodiversity and Conservation Science, The University of Queensland, St Lucia, QLD, Australia; 3 Global Marine Program, Wildlife Conservation Society, Bronx, NY, USA; 4 Sydney Institute for Infectious Diseases, Sydney School of Public Health, The University of Sydney, Camperdown, NSW, Australia; 5 Centre for People Place and Planet, School of Science, Edith Cowan University, Joondalup, WA, Australia; 6 Department of Ecology and Evolutionary Biology, University of California – Irvine, Irvine, CA, USA; 7 Australian Research Council Centre of Excellence for Coral Reef Studies, University of Queensland, St Lucia, QLD, Australia; 8 School of Environment and Science, Griffith University, Nathan, QLD, Australia; 9 Nearshore Habitat Program, Washington State Department of Natural Resources, Olympia, WA, USA; 10 United Nations Environment Programme, Cartagena Convention Secretariat, Kingston, Jamaica; 11 Hawai’i and Palmyra Chapter, The Nature Conservancy, Honolulu, HI, USA; 12 Western Caribbean Department, The Coral Reef Alliance, San Francisco, CA, USA; 13 Science and Conservation, Bush Heritage Australia, Melbourne, VIC, Australia; 14 Centre for Ecosystem Science, University of New South Wales, Sydney, NSW, Australia; 15 Melanesia Program, Wildlife Conservation Society, Suva, Fiji

**Keywords:** Coral reefs, land-based pollution, water-borne disease, systems health, WASH, pathogens

## Abstract

Tropical coastal ecosystems are in decline worldwide due to an increasing suite of human activities, which threaten the biodiversity and human wellbeing that these ecosystems support. One of the major drivers of decline is poor water quality from land-based activities. This review summarises the evidence of impacts to coastal ecosystems, particularly coral reefs, from sediments, nutrients, chemicals and pathogens entering coastal zones through surface and groundwater. We also assess how these pollutants affect the health of coastal human populations through: (1) enhanced transmission of infectious diseases; (2) reduced food availability and nutritional deficit from decline of fisheries associated with degraded habitat; and (3) food poisoning from consumption of contaminated seafood. We use this information to identify opportunities for holistic approaches to integrated watershed management (IWM) that target overlapping drivers of ill-health in downstream coastal ecosystems and people. We demonstrate that appropriate management requires taking a multi-sector, systems approach that accounts for socio-ecological feedbacks, with collaboration required across environmental, agricultural, public health, and water, sanitation and hygiene sectors, as well as across the land–sea interface. Finally, we provide recommendations of key actions for IWM that can help achieve multiple sustainable development goals for both nature and people on coasts.

## Impact Statements

The pollution of water and waterways from land-based human activities has extensive impacts on both human and ecosystem health, contributing to significant global health burdens and loss of critical ecosystem services. Management of pollution is therefore a major focus of multiple sustainable development goals (SDGs) to achieve targets for: zero hunger (SDG 2); good health and well-being (SDG 3); clean water and sanitation (SDG 6); climate action (SDG 13); life below water (SDG 14); and life on land (SDG 15). Despite extensive and complex impacts of poor water quality, pollution control has been highly sectorised and under-resourced, with poor coordination of implementation, often across insufficient scales to realise benefits. This review provides a novel summary of the overlapping impacts of water pollution to downstream public and coastal ecosystem health to support planning and decision-making that benefits a wide range of stakeholders from government, civil society and the private sector. We provide evidence-based suggestions to optimise investments in holistic, integrated watershed management (IWM) to improve water quality and achieve overall systems health, which also provides co-benefits for biodiversity and climate. We also identify the key enabling factors required to coordinate and monitor IWM implementation to achieve desired outcomes. Specifically, the summary of pollution impacts and suggested management strategies provided in this review aim to provide awareness and tools to alleviate impacts to nutrition, water-related disease burdens and food poisoning that arise from poor water quality, which cause devastating economic and health costs disproportionately borne by the poorest countries.

## Introduction

Tropical coastal ecosystems support some of the most diverse and productive environments on Earth and provide millions of people with vital ecosystem goods and services, such as food, livelihoods and coastal protection (Moberg and Folke, 1999; Cesar et al., 2003). However, with over 1.3 billion people in the tropics living within 100 km of coastlines (Sale et al., 2014), coastal ecosystems are becoming increasingly threatened by a suite of local, regional and global human activities, many of which affect water quality (Bellwood et al., 2004; Lotze et al., 2006; Orth et al., 2006). Declining water quality is a primary driver of coastal ecosystem degradation (Crain et al., 2009). Declines in water quality are driven mainly by pollutants from upstream human activities within watersheds flowing into coastal environments and are expected to worsen with increased coastal development and future climate change (Rabalais et al., 2009; He and Silliman, 2019).

Watershed management has received increasing focus as a tool for preserving the health of downstream coastal ecosystems, with research demonstrating critical land–sea linkages for coastal ecosystem health (Carlson et al., 2019; Sahavacharin et al., 2022). Despite the extensive literature and examples of decline, there are few examples of watershed management producing improvements to tropical coastal ecosystem conditions (Wear, 2016). Challenges in achieving measurable success are largely due to the large spatial scale over which interventions often need to be applied within watersheds to adequately address multiple sources of pollution, capacity shortfalls for necessary monitoring, and the temporal lags to detect any changes in water quality and/or ecosystem health within coastal environments (Meals et al., 2010).

Watershed condition also regulates a suite of processes that affect human health and wellbeing, including water filtration, flood management, and the provision of important cultural and recreational services (Jenkins et al., 2018a). Polluted water flowing within watersheds onto coastal environments is a major contributor to global human disease burdens, with poor water quality conservatively estimated to result annually in 1.4 million deaths, 3 million disability-adjusted life years and 12 billion USD in economic losses, a cost disproportionately borne by the poorest countries (Shuval, 2003; Fuller et al., 2022). Yet the influence of watershed management on human health is rarely considered and is largely absent from public health literature (Bunch et al., 2014).

Identifying the overlapping upstream drivers of poor water quality that also create significant risks to public health presents an opportunity to motivate action and leverage long-term and large-scale investments while simultaneously improving coastal ecosystem water quality. By facilitating both human and ecosystem health, watersheds can serve as a focal area for place-based management interventions that serve to promote overall systems health (Cadham et al., 2005; Parkes and Horwitz, 2009; Jenkins et al., 2018b; Jordan and Benson, 2020). Here, we consider systems health as the emergent result of functioning interdependencies, interactions and feedbacks between ecological and socio-cultural settings, behaviour, and physiology, nested across micro-level (e.g., communities of microbes), meso-level (e.g., watersheds) and macro-level (e.g., global climate patterns) domains.

This review aims to: (1) synthesise and summarise the latest science regarding water quality impacts on coastal ecosystems (focused primarily on coral reefs); (2) identify pathways to improve systems health through policy implementation and direct management actions; and (3) provide evidence-based suggestions for strategic investments in watershed interventions across sectors that can help achieve multiple sustainable development goals (SDGs) and other global commitments and targets relating to biodiversity, marine pollution and public health.

## Water quality impacts on coastal ecosystems

The quantity and quality of land-based runoff flowing into adjacent coastal ecosystems is determined by the characteristics of the watershed, such as geology, rainfall, soil type, land cover/vegetation (type and quantity) and slope (Douglas, 1967). There is a large body of evidence that demonstrates how human activities within watersheds alter runoff by removing native vegetation, changing the hydrology, altering microbial communities and adding/increasing pollutants within runoff (e.g., Peters and Meybeck, 2000; Liao et al., 2020).

Several broad pollutant categories are used to describe the pollutants reaching coastal waters from land-based activities. Here, we focus on the following common categories applicable to both human and coastal ecosystem health: sediments, nutrients, persistent organic pollutants (POPs), plastics and microdebris, pathogens, heavy metals, and pharmaceuticals and personal care products (Todd et al., 2010; World Health Organization (WHO), 2016; Kroon et al., 2020). Terrestrially derived sediments, heavy metals and nutrients are naturally transported from soils into coastal environments by ground and surface water, but due to large-scale human activities such as land-clearing (Table 1), the sources and transport into coastal waters has increased drastically, threatening over 30% of coral reefs globally (Andrello et al., 2021). POPs are synthetic organic chemicals that can persist in soils and water and bioaccumulate in organisms. POPs are widely produced across industries (Table 1) both intentionally, such as some insecticides, and unintentionally as by-products, such as dioxins (Weber et al., 2011). Other synthetic pollutants include the nonorganic plastics and microdebris, which can flow into coastal waters from numerous human sources (Table 1) such as trash, litter and weathering of materials like tires (Smith et al., 2018; Macleod et al., 2021). Pathogens are disease-causing microbes and can naturally exist in coastal water and organisms but can also be introduced from land-based sources such as sewage (Table 1). Pharmaceuticals include chemicals used for personal, agricultural or animal health, such as antibiotics, while personal care products include chemicals generally used for cosmetic reasons, such as shampoos and moisturisers (Boxall et al., 2012).

**Table 1.** Key references documenting global/regional linkages between human activities within watersheds and elevated levels of pollutants in runoff to coastal ecosystems

Human watershed activity	Pollutant	Key references
Agriculture	Sediments Nutrients Persistent organic pollutants (e.g., organophosphates and organochlorides in pesticides) Heavy metals (e.g., copper in fertilisers, mercury in fungicides) Pharmaceuticals (e.g., antibiotics) Plastics and microdebris	van Dam et al., 2011; Thorburn et al., 2013; Kroon et al., 2014; MacLeod et al., 2021
Livestock and invasive ungulates	Sediments Nutrients Pathogens (e.g., zoonotic virus/bacteria) Heavy metals (e.g., copper from livestock feed) Pharmaceuticals (e.g., antibiotics) Plastics and microdebris	Agouridis et al., 2005; McDowell and Wilcock, 2008; Todd et al., 2010; Bartley et al., 2014
Aquaculture	Nutrients Persistent organic pollutants (e.g., organotin in molluscicides) Heavy metals (e.g., copper in algaecides) Pharmaceuticals (e.g., antibiotics) Plastics and microdebris	Gräslund and Bengtsson, 2001; Primavera, 2006; Lusher et al., 2017; Wang et al., 2020
Deforestation and burning	Sediments Nutrients Persistent organic pollutants (e.g., polycyclic aromatic hydrocarbons from burning)	Sundarambal et al., 2010; Todd et al., 2010; Suárez‐Castro et al., 2021
Urban development (surface hardening and channel modification)	Sediments	Freeman et al., 2007; Kroon et al., 2014; McGrane, 2016
Mining (including gravel extraction)	Sediments Nutrients Persistent organic pollutants (e.g., polycyclic aromatic hydrocarbons from coal mining) Heavy metals (e.g., lead, nickel, mercury)	Kondolf, 1994; Ahrens and Morrisey, 2005; Todd et al., 2010; van Dam et al., 2011; Shumway, 2020
Wastewater (sewage, domestic, industrial, and storm water)	Sediments Nutrients Pathogens (e.g., water-associated bacteria/virus) Persistent organic pollutants (e.g., oil hydrocarbons from urban storm water) Heavy metals (e.g., tin from industrial wastewater) Pharmaceuticals and personal care products (e.g., antibiotics, psychotropic drugs, and cosmetics) Plastics and microdebris	Loya, 2004; Todd et al., 2010; van Dam et al., 2011; Kroon et al., 2014; Wear and Thurber, 2015; Boucher and Friot, 2017; Littman et al., 2020; Tuholske et al., 2021; Wear et al., 2021

**Figure 1.**
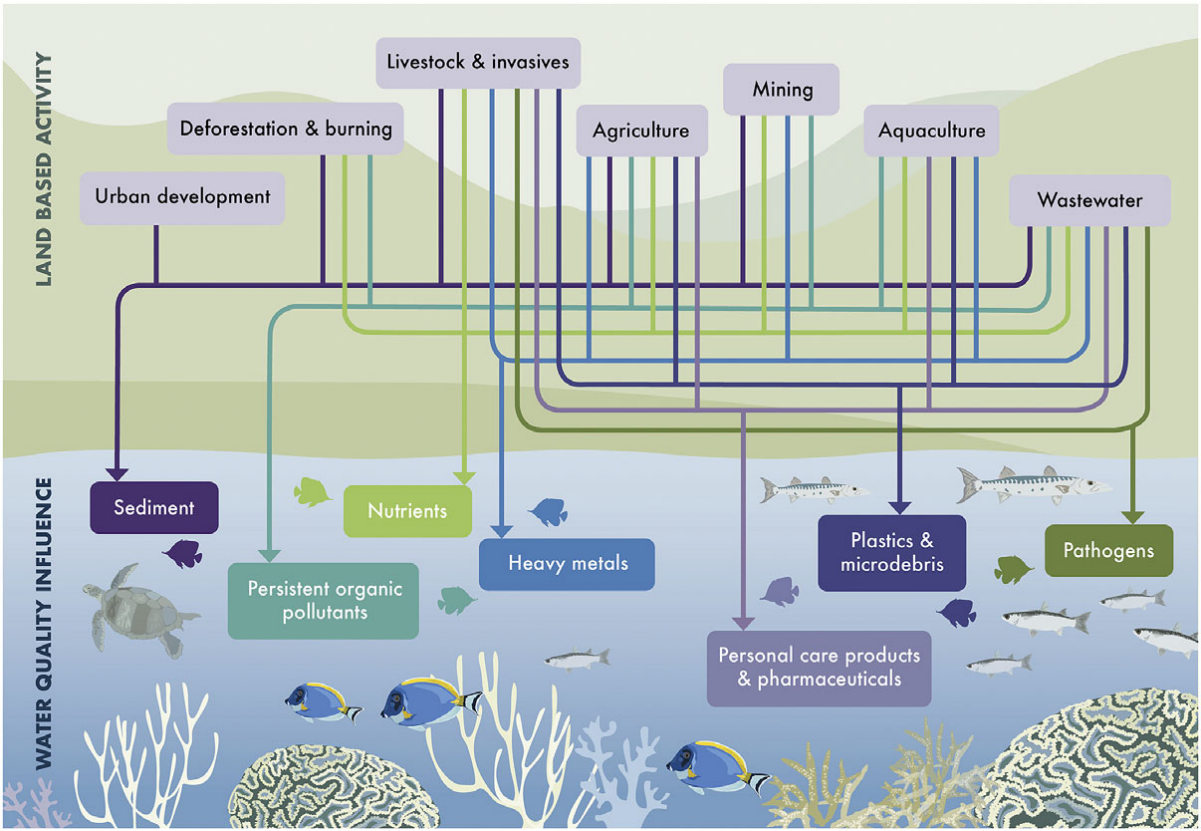
Diagram depicting flow of impacts from key land-based activities on water quality properties that reach coral reef ecosystems.

The primary land-based activities creating these pollutants and driving global declines in coastal water quality are land clearing, poor food production practices, urban development, mining and poor wastewater management (domestic and industrial) (Lu et al., 2018). These human activities erode or release pollutants such as sediment, metals, pathogens and nutrients into surface and groundwater, which are then transported downstream to coastal environments (Crain et al., 2009; Amato et al., 2016). The flow of impacts from human activities within watersheds to coastal ecosystems is summarised below (Figure 1).

As outlined in Table 1, pollutants can have multiple sources that can make it difficult to pinpoint which activity in a watershed is having the greatest impact on coastal ecosystems. For example, nutrients and sediments can originate from both wastewater pollution and agricultural runoff (Figure 1). Similarly, pharmaceuticals and personal care products can originate from cosmetics and medications used domestically as well as from medications used in agriculture (Table 1). In addition to the complexity of sources and types of pollutants, synergistic impacts and interactions occur when multiple pollutants are present at elevated levels, which can exacerbate the degradation of coastal ecosystems and harm associated organisms (Lu et al., 2018; Huang et al., 2021). Synergistic impacts and interactions also occur when pollutants are present with other stressors, such as climate change, disease, invasive species and overfishing. We focus on synergistic interactions on coral reefs, given the large body of research.

Herbivory is an important ecological process within coral reef ecosystems and can have complex and synergistic interactions with poor water quality (Table 2; Mumby et al., 2007). For example, in reefs with combined exposure to poor water quality and few herbivores, macroalgae and sediment-laden turfs can replace live coral as the dominant benthos (McField et al., 2020, 2022). Sea level rise and climate-driven ocean warming are predicted to increase the sensitivity of coral reef ecosystems to poor water quality. Land-based pollution can lower the threshold for thermal stress and increase coral sensitivity to infection, resulting in increased bleaching (Fisher et al., 2019), coral mortality (Claar et al., 2020) and outbreaks of disease on coral reefs (Vega-Thurber et al., 2020). Corals that bleach from thermal stress also have reduced capacity to cope with sediment pollution (Bessell-Browne et al., 2017). Nutrient pollution can result in brittle corals that are less resilient to the impacts of climate change, such as sea level rise and the increased severity and frequency of cyclones (Table 2; Rice et al., 2020). Improving water quality through management of human activities within watersheds can therefore improve the resilience of corals to global impacts such as climate change.

**Table 2.** Impacts of poor water quality on humans, coral reefs, and coral reef organisms categorised by pollutant type, with key references indicated for further information

Pollutant	Impacts to health			
Terrestrially derived sediment	**Humans**		**Human populations**	**Key references**
	Increased cost and complexity of water treatment.		► Can lead to inadequate coverage of treated water and increased time/cost accessing safe water sources.	World Health Organization (WHO), 2012, 2016; Price and Heberling, 2018; Albert et al., 2021
	Increased risk of water-related diseases in humans.		► Increased mortality and comorbidity, healthcare burdens.	World Health Organization (WHO), 2012, 2016; Jenkins et al., 2016; Herrera et al., 2017; Albert et al., 2021
	Change in aesthetic of water for human use.		► Loss of cultural and spiritual values, reduced tourism benefits.	World Health Organization (WHO), 2012; 2016; Landrigan et al., 2020
	**Coral reef organisms**	**Coral reef ecosystems**	**Human populations**	**Key references**
	Reduced fertilisation and settlement for coral and reef building species. Reduced coral growth rate, colony size, and photosynthetic yield. Partial mortality.	► Reduced reef accretion and coral cover reduces habitat complexity and the capacity of coral reef ecosystems to recover from disturbances.	► Reduced coastal protection, tourism benefits, fisheries services, and may lead to human health impacts from reduced nutrition.	Rogers, 1990; Van Woesik and Done, 1997; Gilmour, 1999; Wesseling et al., 2001; Philipp and Fabricius, 2003; Fabricius, 2005; Bessell-Browne et al., 2017; Ricardo et al., 2018; Jones et al., 2019
	Reduced coral species richness and altered coral complexity and community composition.	► Reduced ecosystem diversity and habitat complexity, which impacts the capacity of coral reef ecosystems to recover from disturbances.		Rogers, 1990; Edinger et al., 1998; West and van Woesik, 2001; Golbuu et al., 2008; van der Meij et al., 2010
	Suppression of herbivory by reef fish. Reduced abundance of herbivorous fish species. Accumulation in algal turfs.	► Proliferation of coral-inhibiting algae, reducing coral cover and the capacity of coral reef ecosystems to recover from disturbances. Reduced structure and nutrition also leads to reduced fish populations and diversity.		Wenger et al., 2015; Moustaka et al., 2018; Tebbett and Bellwood, 2019; Wenger et al., 2020
	Extended larval development and reduced settlement of fish. Gill damage and mortality of fish. Increased susceptibility to disease of larval fish. Reduced foraging ability. Reduced fish species richness.	► Reduced fish recruitment, biomass and diversity, which impacts capacity of reef ecosystems to recover from disturbance.	► Reduced tourism benefits, fisheries services, and may lead to human health impacts from reduced nutrition.	Hess et al., 2015; Wenger et al., 2015; Moustaka et al., 2018
Nutrients (organic and inorganic)*	**Humans**		**Human populations**	**Key references**
	Severe health impacts for human infants through consumption of contaminated water.		► Increased mortality and comorbidity.	World Health Organization (WHO), 2016
	**Coral reef organisms**	**Coral reef ecosystems**	**Human populations**	**Key references**
	Reduced fertilisation, settlement and reproductive success of corals. Reduced coral growth rate Partial or complete mortality of corals	► Reduced reef accretion and coral cover reduces habitat complexity and the capacity of coral reef ecosystems to recover from disturbances.	► Reduced coastal protection, fisheries services, and may lead to health impacts from reduced nutrition.	Harrison and Ward, 2001; Koop et al., 2001; Cox and Ward, 2002; Loya, 2004; Todd et al., 2010; Weber et al., 2012
	Reduced calcification and coral skeletal density. Increased macrobioeroder density in corals.	► Brittle corals that are more susceptible to breaking and erosion. This reduces the capacity to recover from disturbances.	► Reduced coastal protection.	Edinger et al., 2000; Koop et al., 2001; Fabricius, 2005; Le Grand and Fabricius, 2011; Rice et al., 2020
	Increased algal growth.	► Proliferation of coral-inhibiting algae under reduced herbivory, reducing coral cover and the capacity of coral reef ecosystems to recover from disturbances.	► Reduced coastal protection, fisheries services, and may lead to health impacts from reduced nutrition	McManus et al., 2000; Lapointe et al., 2011
	Coral disease.	► Potential reductions in the composition, abundance, and ultimately the accretion of coral. Limited information at present.	► Reduced coastal protection.	Redding et al., 2013
Pathogens	**Humans**		**Human populations**	**Key references**
	Increased risk of water-related diseases in humans.		► Increased mortality and comorbidity, healthcare burdens.	Fleming et al., 2006; Sindermann, 2006; Lau et al., 2010; World Health Organization (WHO), 2016, 2019; Lamb et al., 2017
	**Coral reef organisms**	**Coral reef ecosystems**	**Human populations**	**Key references**
	Coral disease.	► Potential reductions in the composition, abundance, and ultimately the accretion of coral. Potential for corals to act as vectors or reservoirs of human pathogens. Limited information at present.	► Reduced coastal protection services. Potential increase in health burdens from waterborne infectious disease.	Sutherland et al., 2011
	Increased pathogenic microbiota on fish gills and shellfish.	► Potential outbreak of disease and reductions in fish recruitment. Limited information at present.	► Potentially reduced fisheries services, and may lead to health impacts from reduced nutrition and increase in food poisoning.	Shuval, 2003; Hess et al., 2015; World Health Organization (WHO), 2015
Persistent organic pollutants (POPs)	**Humans**		**Human populations**	**Key references**
	Severe health impacts (e.g., cardiovascular disease, toxicity and developmental defects) through consumption of contaminated water.		► Increased mortality and comorbidity, reduced schooling attendance, healthcare burdens.	World Health Organization (WHO), 2016; Landrigan et al., 2020; Müller et al., 2020
	Promotion of antifungal resistant pathogens.		► Limited information at present.	World Health Organization (WHO), 2014; O’Neill, 2016; Woolhouse et al., 2016
	Potential health impacts from immune and endocrine disruption.			World Health Organization (WHO), 2016
	Offensive odour in water.		► Loss of cultural and spiritual values, reduced tourism benefits.	World Health Organization (WHO), 2012, 2016;
	**Coral reef organisms**	**Coral reef ecosystems**	**Human populations**	**Key references**
	Reduced fertilisation, settlement, and development of corals and coral reef organisms. Accumulation in coral and coral reef organisms. Reduced photosynthetic efficiency, chlorophyll concentration, and symbiont density in corals. Reduced growth of coral reef building organisms. Partial and complete mortality of coral and coral reef organisms. Coral bleaching.	► Reduced coral cover and reef accretion. Ultimately reduces coral reef ecosystem capacity to recover from disturbances.	► Reduced coastal protection, fisheries services, and may lead to health impacts from reduced nutrition.	Todd et al., 2010; Turner and Renegar, 2017; Ranjbar Jafarabadi et al., 2018; Kroon et al., 2020; Nalley et al., 2021
	Olfactory impairment in fish.	► Limited information on chronic exposure at present. Could alter fish population dynamics and leave coral reef organism’s vulnerable to additional stressors.	► Potentially reduced fisheries services which may lead to health impacts from reduced nutrition.	Wenger et al., 2015
	Endocrine disruption in fish and other coral reef organisms.			
	Immuno-suppression in fish.			
	Accumulation in fish and molluscs.		► Severe disease and impacts to developing infants through consumption of contaminated seafood.	Landrigan et al., 2020
Heavy metals	**Humans**		**Human populations**	**Key references**
	Severe health impacts (e.g., developmental defects and toxicity) through contact with or consumption of contaminated water.		► Increased mortality and comorbidity, reduced schooling attendance, healthcare burdens.	World Health Organization (WHO), 2016; Rehman et al., 2018; Landrigan et al., 2020
	Inhibition of biological sewage treatment.		► Can lead to inadequate coverage of treated water and increased time/cost accessing safe water sources.	World Health Organization (WHO), 2016
	**Coral reef organisms**	**Coral reef ecosystems**	**Human populations**	**Key references**
	Reduced fertilisation, settlement, and development of corals. Coral bleaching. Reduced chlorophyll concentration and symbiont density in corals. Partial and complete mortality of corals.	► Reduced coral cover and reef accretion. Ultimately reduces coral reef ecosystem capacity to recover from disturbances.	► Reduced coastal protection, fisheries services, and may lead to health impacts from reduced nutrition.	Negri et al., 2002; Nalley et al., 2021
	Embryo malformation and reduced hatching success in fish. Olfactory impairment and behavioural changes in fish.	► Fish larvae and new recruits are potentially more prone to predation. Limited information on chronic and lower levels of exposure at present.	► Reduced fisheries services and may lead to health impacts from reduced nutrition. Various severe health impacts from seafood consumption	Wenger et al., 2015
	Immuno-suppression in fish. Accumulation in fish and molluscs.	► Increased disease and death rates in fish.		Bosch et al., 2016; Landrigan et al., 2020
	Endocrine disruption of fish reproduction.			
Personal care products and pharmaceuticals	**Humans**		**Human populations**	**Key references**
	Promotion of antimicrobial resistant water related pathogens.		► Increased mortality and comorbidity, healthcare burdens.	World Health Organization (WHO), 2014; Woolhouse et al., 2016
	Potential endocrine disruption of human development and immune systems.		► Limited information at present.	World Health Organization (WHO), 2016
	**Coral reef organisms**	**Coral reef ecosystems**	**Human populations**	**Key references**
	Endocrine disruption of coral fecundity. Endocrine disruption of development and/or growth of coral and coral reef organisms. Reduced tissue regeneration in coral Mortality of coral Coral bleaching	► May lead to reductions in the composition, abundance, and ultimately the accretion of coral. Limited information at present	► Potentially reduced coastal protection and fisheries services.	Tarrant et al., 2004; Wear and Thurber, 2015; Downs et al., 2016; Watkins and Sallach, 2021
	Endocrine disruption of development and/or growth in fish Altered predator–prey interactions and aggressive behaviour of fish.	► May lead to changes in fish population dynamics and communities	► Potentially reduced fisheries services.	Wenger et al., 2015
	DNA alterations and reduced reproduction and development in crustaceans.	► May lead to changes in population dynamics of commercially harvested species	► Potentially reduced fisheries services.	Garcia et al., 2014; Maranho et al., 2014
	Growth inhibition in algae.	► May lead to changes in fish population dynamics and communities, potentially causing trophic cascades in reefs	► Potentially reduced fisheries services.	Aguirre-Martínez et al., 2015
Plastic and microdebris	**Humans**		**Human populations**	**Key references**
	Potential impacts to health (e.g., infertility and damage to the nervous system). Increase favourable conditions for pathogens.		► Limited information at present. Potential increases in disease burden associated with contaminated water. Plastic consumption potentially leading to health hazards.	Zettler et al., 2013; Galloway, 2015; Kirstein et al., 2016
	**Coral reef organisms**	**Coral reef ecosystems**	**Human populations**	**Key references**
	Reduced reproduction and growth Disease, bleaching and tissue necrosis	► Limited information at present. May lead to reductions in the composition, abundance, and ultimately the accretion of coral.	► Limited information at present. Potentially reduced coastal protection services and reduced fisheries services.	Todd et al., 2010; Lamb et al., 2018; Huang et al., 2021
	Enhanced transport of other contaminants to corals.	► Limited information at present. May lead to a range of contaminant-specific impacts, such as reduced coral cover and reef accretion from sediment.		Littman et al., 2020
	Accumulation in fish, molluscs, and other reef associated organisms. Enhanced transport of pathogens and other contaminants to fish, molluscs, and other reef associated organisms.	► Limited information at present. May lead to changes in population dynamics and communities in marine ecosystems.	► Limited information at present. Potential increases in disease burden associated with contaminated seafood consumption. Plastic consumption potentially leading to health hazards.	Smith et al., 2018; Littman et al., 2020; Wu and Seebacher, 2020; Zhang et al., 2022

* Contaminant dynamics are complex, with different impacts and response curves observed even between contaminants in the same group (e.g., different heavy metals generate different impacts, different types of nutrients generate different impacts). Different levels of exposure also generate different responses, with some nutrient species generating positive responses under certain exposure levels. Impacts reported here are a general summary of known impacts from the introduction of each contaminant group at harmful levels observed in the environment.

## Water quality impacts on human health

Many of the same drivers of declines in water quality and aquatic biodiversity, such as watershed deforestation, forest fragmentation on riverbanks and poor coverage of sanitation services, are also associated with human health impacts (Table 2). Impacts to humans from poor water quality include enhanced transmission of disease through polluted water and waterways, nutrition deficits from fisheries decline and chronic illness, and food poisoning from the contamination of important aquatic foods (Shuval, 2003; World Health Organization (WHO), 2015; Chase and Ngure, 2016). Over a million people die each year from water-related diseases, and at least 50% of these deaths are children and attributable to microbial intestinal infections (Kovacs et al., 2015). Water related diseases such as diarrhoea are major contributors to global disease burdens, causing 8% of all deaths in children under the age of 5 years largely due to inadequate drinking-water quality (United Nations Inter-Agency Group for Child Mortality Estimation (UN IGME), 2019; World Health Organization (WHO) and United Nations Children’s Fund (UNICEF), 2021). Persistent endemicity and explosive outbreaks of water-related disease are often fuelled by interacting environmental factors related to climate change, land use and changing social conditions (Cann et al., 2013; Prüss-Ustün et al., 2019). Water-related illness and travel associated with accessing safe water sources also contributes to reduced socioeconomic outcomes, such as reduced school attendance and gender equity (Fisher, 2008; Sorenson et al., 2011).

Communities reliant on surface and groundwater sources for drinking, bathing and household cleaning water are most at risk to water-related diseases and exposure to pollutants of emerging concern, particularly in tropical environments (Ragosta et al., 2011; World Health Organization (WHO), 2016; Herrera et al., 2017). Climate change is predicted to further increase global disease burdens by altering water-related disease dynamics (Semenza, 2020). Changes in rainfall and temperature will threaten water security, enhance pathogen survival and virulence, and increase exposure to contaminated water through multiple pathways, including flooding (Hofstra, 2011; Levy et al., 2018). Rates of diarrhoea are predicted to increase under warmer and/or wetter conditions, with 1°C of warming predicted to increase diarrhoeal disease by 5% in developing countries (Singh et al., 2001).

Although water-related diseases are more often associated with exposure on land and freshwater, polluted seawater also presents a significant risk to human health. An estimated 180 million cases of upper respiratory disease and gastroenteritis occur each year due to humans bathing in polluted ocean waters or ingesting contaminated seafood, while around 4 million cases (and 40 thousand deaths) of infectious hepatitis A and E (HAV/HEV) occur annually from contaminated seafood from polluted coastal waters (Shuval, 2003; World Health Organization (WHO), 2015). Additionally, seafood contaminated with methylmercury and polychlorinated biphenyls can cause cardiovascular diseases in humans as well as severe impacts to infants in utero (Landrigan et al., 2020). The impacts of polluted seawater create a huge social and economic cost to communities, with pathogens in ocean pollution causing an estimated $19.4 billion (2022 USD) in economic losses annually because of their direct impacts on humans alone (Shuval, 2003).

Microplastics and debris found in wastewater pollution can also form a unique microbial community that is distinct from the surrounding water (Zettler et al., 2013). The microbial community on plastic can include pathogenic microorganisms, such as *Vibrio* spp., that cause infections through contaminated water or seafood consumption (Zettler et al., 2013; Kirstein et al., 2016). In the case of some zoonotic parasitic microbes that cause illness in aquatic wildlife and illness in humans from shellfish consumption, counts of the microbes are higher on plastics than in surrounding water (Zhang et al., 2022). Plastics therefore potentially create a novel habitat for pathogens to be concentrated and dispersed beyond their typical range, as floating plastics can travel longer distances than natural substrates (e.g., wood and macroalgae), and sinking microplastics are readily ingested by filter-feeding shellfish (Zettler et al., 2013; Littman et al., 2020; Zhang et al., 2022).

Polluted coastal ecosystems also affect the health of coastal human populations through fisheries decline (Hicks et al., 2019; Li et al., 2019). Millions of people depend on tropical coastal fisheries for essential protein and micro-nutrients (Kawarazuka and Béné, 2010; Teh et al., 2013). More than 10% of the global population is likely to face micronutrient and fatty acid deficiencies if the current trajectories of fisheries decline continue, especially in the developing nations at the Equator (Golden et al., 2016). In addition, individuals already experiencing chronic health effects due to repeated exposure to pathogens will have nutrient absorption challenges, further exacerbating any micronutrient deficiencies from declining fisheries (Chase and Ngure, 2016). Better recognition of the economic and human health costs resulting from pollution impacts is critical for prioritising action and leveraging the necessary cross-sectoral partnerships and resources required for managing pollution at appropriate scales.

## Systems approaches to watershed management

There are an array of site-based management interventions that can be implemented at nested scales within watersheds to improve water quality (Liu et al., 2017; Richmond et al., 2019; Leder et al., 2021). Mitigation efforts typically include policy instruments and place-based interventions.

Policy instruments, such as regulations or market-based incentives, can be applied at any scale and are not necessarily spatially bound within watersheds or aimed at specific watersheds. For example, the implementation of policy instruments can control, reduce and/or prevent pollution through improved use, transport, storage and disposal of chemicals (Taylor et al., 2012; Olmstead and Zheng, 2021) and nutrients (UNEP, 2012). Policy instruments can also initiate the implementation of soil conservation and erosion/runoff control strategies, such as maintaining riparian buffer zones by legislating mangrove protection (Richmond et al., 2019).

**Figure 2.**
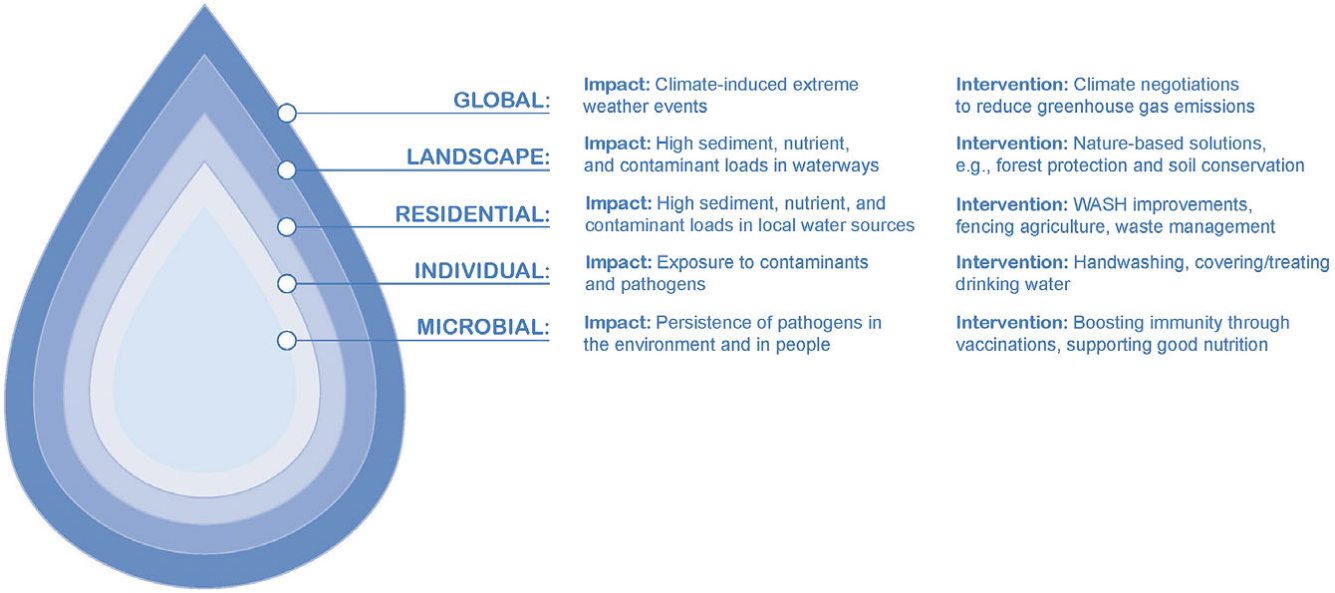
Nested scales of watershed processes.

Place-based interventions are specifically applied at a range of scales, from landscape, residential, down to individual and microbial scales (Figure 2). Traditionally, human health focused place-based interventions have been targeted at a residential and individual scale, through the application of water, sanitation and hygiene (WASH) infrastructure improvements or behaviour change campaigns (World Health Organization (WHO), 2016; World Health Organization (WHO) and United Nations Children’s Fund (UNICEF), 2021). However, there is now substantial evidence that landscape scale interventions could deliver significant human health outcomes, while also protecting ecosystem health. For example, a study involving 35 developing countries found that higher upstream tree cover in watersheds was associated with a lower probability of childhood diarrhoeal disease downstream (Herrera et al., 2017). In Hawai'i Ragosta et al. (2011) demonstrated that higher riparian canopy cover was associated with lower *Enterococcus* concentrations in stream water. New genomics research is beginning to reveal how more intact ecosystems, from the watershed to the individual organism scale, are more likely to carry lower pathogen loads (Hess et al., 2015; Shore-Maggio et al., 2018; Bass et al., 2019). Coastal ecosystems also play a key role in regulating disease risk in the marine environment, with a recent study showing that when seagrass meadows are present, there are 50% fewer potentially pathogenic bacteria capable of causing disease in humans and aquatic organisms (Lamb et al., 2017). However, coastal ecosystems themselves are vulnerable to high levels of pollution (Crain et al., 2009; Wear, 2016; Turschwell et al., 2021), underscoring the importance of implementing a system-wide approach when managing watersheds.

Despite the recognition that pollution is one of the greatest threats facing coral reef ecosystems (Burke et al., 2011; Andrello et al., 2021), there are limited examples of water quality management associated with successful recovery of coral reef ecosystems, and of those, the management interventions have primarily only tackled pollution arising from point-source pollution (Birkeland et al., 2013; Reef Resilience Network, 2021). Designing and measuring the effectiveness of policy instruments for water quality management is difficult due to lack of compliance and information on contaminant thresholds and monitoring (Taylor et al., 2012; Olmstead and Zheng, 2021). Place-based interventions are often impeded by difficulties in engaging stakeholders, lack of systematic/transparent planning, and funding shortfalls (Jupiter et al., 2017; Ayala-Orozco et al., 2018). For example, where stakeholders are not effectively engaged, interventions can be hindered by divergent visions, interests, and tensions within and between sectors (Ayala-Orozco et al., 2018). Lack of engagement can also limit buy-in and uptake of interventions by groups (Oteros-Rozas et al., 2015; Mitchell et al., 2022). Lack of systematic/transparent planning and evaluation can generate a lack of trust, accountability and credibility from the perspective of stakeholders (Ayala-Orozco et al., 2018), and lead to missed opportunities for effective action (Jupiter et al., 2017; Beer et al., 2020). Funding shortfalls and lack of personnel prohibit action at the scale and duration required (Ayala-Orozco et al., 2018; Beer et al., 2020). Interventions for nonpoint source pollution can be particularly challenging as pollution loading is difficult to estimate and is often attributable to many stakeholders and sectors beholden to different regulations (Shortle and Horan, 2001).

Kāneʻohe Bay in Hawaii is a commonly cited case-study of point-source pollution (sewage) management for coral reef ecosystems resulting in a rarely seen recovery from an algal dominated back to a coral dominated state (Bahr et al., 2015). More recent successes include recovery of coral reef ecosystems within Faga’alu Bay in American Samoa and Molokaʻi in Hawai‘i, where harmful runoff from the upstream quarry activities (Samoa) and invasive ungulate species (Hawaiʻi) were managed through targeted watershed interventions (Vargas-Ángel and Huntington, 2020). Both regions’ intervention strategies required large and costly monitoring efforts to observe success, and both observed setbacks in recovery trajectories due to external disturbances (e.g., storm waves and bleaching; Bahr et al., 2015; Vargas-Ángel and Huntington, 2020).

### Watershed case study 1: Watershed interventions for systems health in Fiji

Low coverage of properly treated drinking water and sanitation in remote areas of Fiji leaves communities heavily reliant on the safety and security of unprotected water sources and vulnerable to water-related diseases. Severe outbreaks of water-related infectious diseases, such as leptospirosis, typhoid and dengue (hereafter LTD), are common. LTD cases and associated syndromes are correlated with environmental conditions, with large outbreaks typically occurring following heavy rainfall and flooding (Lau et al., 2010; Nelson et al., 2022), with increased severity within degraded watersheds (Jenkins et al., 2016).

Coastal and freshwater ecosystems are also threatened by degraded watersheds in Fiji, with decreased fish, coral and seagrass cover seen downstream of cleared and developed watersheds due to the runoff of harmful pollutants (Jenkins et al., 2010; Brown et al., 2017; McKenzie and Yoshida, 2020). These ecosystems support the livelihoods, nutrition and incomes of many rural communities (Mangubhai et al., 2018).

The Watershed Interventions for Systems Health in Fiji (WISH Fiji) project aims to address these overlapping problems through a collaborative effort between government, academic and non-governmental organisations (NGO) partners. Project collaborators are co-designing targeted ‘up-stream’ interventions implemented across various nested scales (Figure 2) with local communities to prevent, detect and respond to LTDs, in addition to mitigating degradation of downstream resources and ecosystems (McFarlane et al., 2019). In doing so, the WISH Fiji project aims to transform both environmental and public health action from reactive to preventative, and improve the overall health of the system to maintain integrity against LTD and natural disasters.

### Watershed case study 2: Wastewater management in Roatan, Honduras

Roatan Island, in the Bay Islands of Honduras, is bordered by coral reef ecosystems that attract over a million tourists into the region. Provisioning unpolluted runoff from watersheds is essential to maintaining the health of these ecosystems, but also to protect the health of Roatan communities and tourists. However, limited wastewater treatment on the island resulted in discharge of untreated or inadequately treated wastewater directly onto coral reef ecosystems. Local ecological knowledge linked this wastewater runoff to outbreaks of water-related infectious disease in both humans and corals in the region, which raised fears of impacts on tourism (the main source of income in Roatan).

To combat both the human health and ecosystem impacts of untreated wastewater discharge, a collaboration between government, conservation groups and water associations identified the need for a community wastewater treatment plant (WWTP) and water quality program in West End, Roatan. The West End WWTP was then built in 2011 and has since been connected to 99% of accessible homes and businesses in the area.

Critically, a water quality laboratory led by the Bay Islands Conservation Association was also built to enable testing of marine water downstream of the WWTP, allowing significant improvements in water quality to be observed. Within 7 years of the WWTP installation, the public beach downstream passed the United States EPA safe swimming standards for *Enterococcus*, a bacteria which can cause a variety of infections and is associated with faecal contamination. The beach has since been awarded an Ecological Blue Flag certification that validates the areas as safe for tourists. Improved metrics for coral reef ecosystem health were also observed, likely as a result of improved water quality (Coral Reef Alliance, 2020).

## Key enabling factors

### Cross-sectoral coordination and integrated governance

Managing watersheds offers numerous opportunities to address systems health challenges linked to achievement of multiple SDGs (Jenkins et al., 2018a), but simultaneously tackling multiple objectives requires coordination and integrated governance. Cross-sectoral collaborations can create a more holistic understanding of the watershed system and the breadth of its impacts across sectors (Parkes et al., 2010). This holistic understanding can improve the efficiency of integrated watershed management (IWM) by targeting multiple problems at once, creating the potential for win–win scenarios for both coastal ecosystem health and human health (Jupiter et al., 2014; Jenkins and Jupiter, 2015).

The success of cross-sectoral coordination and governance relies on careful participatory engagement and integrated policy development and implementation (Olsen and Christie, 2000; Lane, 2008). Decision-making should be developed through engagement with a wide range of stakeholders and resource users at multiple scales, improving coordination between divisions that may typically focus on the coastline or in specific sectors (Wang et al., 2016). Care should be taken to incorporate information from multiple knowledge systems in planning and practice to ensure alignment with local values and objectives (Tengö et al., 2014). Engagement should capture the diversity of land and water use practices, needs, goals and potential conflicts across sectors, and ensure that all involvement is participatory, transparent, accountable and culturally appropriate (Jupiter et al., 2014; Richmond et al., 2019).

Managing watersheds for systems health often requires coordination across multiple jurisdictions and administrative units that operate within and beyond watershed boundaries. Watershed governance is thus complicated by the mismatched boundaries of biophysical processes operating within drainage basins and jurisdictional boundaries of administrative systems responsible for land use policy implementation and health systems surveillance and delivery (Davidson and De Loë, 2014). Polycentric and collaborative governance approaches, particularly those involving Indigenous peoples and local communities, are appropriate in this context to bridge across sectors and jurisdictional levels and address watershed systems issues at appropriate scales (e.g., Huitema et al., 2009; Morrison, 2017). Watershed management across multiple agencies and organisations can be coordinated by specific institutions that can serve as bridging organisations, such as catchment authorities, which operate most effectively when they have legislated mandates and operating budgets (Parkes et al., 2010; Davidson and De Loë, 2014).

Critically, integrated policy needs to be developed based on a good understanding of the connections among systems so that evidence-based predictions and decisions can be made about how any interventions may influence outcomes in multiple sectors (Álvarez-Romero et al., 2015). It is essential to consider any potential trade-off scenarios wherein mutual benefits are not shared between sectors, or one sector may even be exposed to more harm. For example, the construction or restoration of wetlands for improving water quality and ecosystem health may have unintended consequences for mosquito-borne disease risk (Malan et al., 2009; Horwitz and Finlayson, 2011); and the installation of dams and weirs for improving water security and sediment pollution may have unintended consequences for freshwater ecosystems and fisheries (Dudgeon et al., 2006; Kroon et al., 2014). Having a wide range of informed stakeholders sharing resources and taking an integrated approach will assist in buffering this risk and create more effective and proactive governance wherein benefits across sectors are optimised.

### Sustainable financing

Improving water quality through upstream interventions is expensive and requires sustained investment (Muchapondwa et al., 2018). There is often a long lag time between implementing interventions and observing improvements in metrics of ecosystem and public health, while success can also be obscured by other disturbances, such as cyclones and coral bleaching (Richmond et al., 2019). Delays in realising anticipated benefits create disincentives for long-term action when program and policy targets require short-term results.

Water and watershed funds are a common financing tool used in various geographies globally to ensure a sustained source of funding (The Nature Conservancy (TNC) and Goldman, 2009; Kauffman, 2014). These funds are often resourced through voluntary contributions of donors and water users, such as utility companies and farmers, which are then used to pay for and support upstream strategies to conserve the quality and security of water sources. Boards may invest the funding directly or use grants to identify and develop critical intervention strategies (The Nature Conservancy (TNC) and Goldman, 2009). Linking the needs of downstream water users with upstream communities and land users allows the funds to provide a low-cost and sustainable financing method of maintaining clean and regular water supply (The Nature Conservancy (TNC) and Goldman, 2009).

Examples of successful water funds are mainly from temperate regions and exclude marine ecosystems, such as the Latin American Water Funds Partnership (LAWFP). LAWFP is an agreement between a consortium of international NGOs to enhance and preserve water security in Latin America and supports 25 water funds across nine countries with varying water management goals and local funding bodies (Bremer et al., 2016). In total, LAWFP supported water funds are managing over 227,000 ha of land, potentially benefiting 89 million people, and have leveraged over $205 million USD in resources. Many funds prioritise not only water infrastructure management for humans, but also the use of nature-based solutions as a means to preserve the health of aquatic ecosystems (Kauffman, 2014). However, as with many water funds (and conservation efforts), there have been limited measurements of the outcomes or baselines to fully perceive the benefits of these funds (Bremer et al., 2016).

The availability of local sources of funding for sustainable financing of a water or watershed fund will vary from region to region as beneficiaries vary. Not all communities and industries pay for water use: under these circumstances, it may be feasible to develop business cases for investment based on foregone healthcare and productivity costs if watershed improvements prevent people from getting sick. Key to developing these business plans is first assessing how much disease risk can be reduced by a portfolio of management interventions and balancing the wide range of savings in foregone costs (healthcare, missed work and education, tourism impacts) against annual investment needs. Considerations also need to be taken for the potential benefits from buffering against the influence of climate change on disease.

Various other types of conservation and climate change financing can additionally or alternatively support watershed management financing. For example, in some coral reef areas, payment for ecosystem services schemes have also been proposed as a way for downstream resource users to incentivise upstream resources users to manage water quality (Goldman-Benner et al., 2012; Peng and Oleson, 2017). Climate financing that supports nature-based solutions is commonly expected to deliver various water services, though evidence shows mixed results on base flow, annual surface runoff and water quality depending on local geographic conditions and the mix of interventions utilised (Vigerstol et al., 2021; de Freitas et al., 2022).

## Conclusions/recommendations

The latest science makes it clear that unplanned development, poor land use, unsustainable agricultural practices and poor wastewater management within watersheds are significant threats to coastal populations and ecosystems. Despite the threats, incentivising improved watershed management practices for the sake of improving water quality for downstream environmental benefits has remained a challenge. In the future, it is recommended that policies and management are designed using systems health approaches that aim to restore water quality to achieve multiple benefits for human and coastal ecosystem health, while facilitating sustainable social and economic development.

Through our review, we identified a series of actionable recommendations to promote holistic approaches to watershed management for systems health (Table 3). These include best practice lessons from existing, IWM programs on: inclusive planning; implementation through cross-sectoral coordination; participatory management; monitoring to identify risks and measure progress of interventions; mobilising resources to sustain long-term action; and sharing information to promote replication and scaling of integrated approaches. To achieve SDG targets by 2030, there is increasing urgency to prioritise these types of management approaches that simultaneously deliver on benefits for nature, people and climate.

**Table 3.** Recommendations for planning, coordinating, monitoring, resourcing and scaling sustained investment in integrated watershed management for systems health

Planning	Ensure engagement of the full range of actors, landowners and beneficiaries within watershed boundaries and provide platforms for transparent, participatory planning and decision-making.
Coordinating	Undertake policy gap analysis and strategic environmental assessments to improve harmonisation and implementation of existing policies
	Engage, strengthen and/or establish multi-sector management authorities (e.g., watershed commissions) with the mandate and resources to coordinate action across marine resource users/managers, logging, mining, agricultural, public health, tourism and WASH sectors.
Monitoring	Undertake risk and resilience assessments to identify main sources of land-based impacts to coastal reef ecosystems, and consider where these risks overlap with risks to public health, especially in the context of future climate change scenarios.
	Conduct research and synthesis to improve the quantity and quality of data available on thresholds and indicators of water quality and impacts on coral reef ecosystems, including integration of indigenous knowledge, citizen science, and private sector, and making the information easily accessible (i.e., through new knowledge management products and an open-source water quality database) to support monitoring and assessment programs.
Resource mobilisation	Develop/enhance sustainable and innovative financing mechanisms, through impact investment and private sector engagement, business case studies and integrated resource mobilisation strategies, to provide the resources required to implement phased, integrated watershed management interventions across nested scales.
Scaling	Develop guidance materials to integrate coral reef ecosystem health into integrated watershed management, public health and WASH planning.
	Document the process of developing and implementing integrated watershed management strategies in order to create communication materials for the broader conservation, WASH and public health communities on best practises and lessons learned, working with regional agencies and mechanisms to upscale.

## Data Availability

No data presented in this review.

## References

[r1] Agouridis CT, Workman SR, Warner RC and Jennings GD (2005) Livestock grazing management impacts on stream water quality: A review. Journal of the American Water Resources Association 41, 591–606.

[r2] Aguirre-Martínez GV, Owuor MA, Garrido-Pérez C, Salamanca MJ, Del Valls TA and Martín-Díaz ML (2015) Are standard tests sensitive enough to evaluate effects of human pharmaceuticals in aquatic biota? Facing changes in research approaches when performing risk assessment of drugs. Chemosphere 120, 75–85. 25000509 10.1016/j.chemosphere.2014.05.087

[r3] Ahrens MJ and Morrisey DJ (2005) Biological effects of unburnt coal in the marine environment. Oceanography and Marine Biology 43, 69–122.

[r4] Albert S, Deering N, Tongi S, Nandy A, Kisi A, Sirikolo M, Maehaka M, Hutley N, Kies-Ryan S and Grinham A (2021) Water quality challenges associated with industrial logging of a karst landscape: Guadalcanal, Solomon Islands. Marine Pollution Bulletin 169, 112506. 34052589 10.1016/j.marpolbul.2021.112506

[r5] Álvarez-Romero JG, Adams VM, Pressey RL, Douglas M, Dale AP, Augé AA, Ball D, Childs J, Digby M, Dobbs R, Gobius N, Hinchley D, Lancaster I, Maughan M and Perdrisat I (2015) Integrated cross-realm planning: A decision-makers’ perspective. Biological Conservation 191, 799–808.

[r6] Amato DW, Bishop JM, Glenn CR, Dulai H and Smith CM (2016) Impact of submarine groundwater discharge on marine water quality and reef biota of Maui. PLoS One 2016, e0165825. 10.1371/journal.pone.0165825 PMC5094668 27812171

[r7] Andrello M, Darling ES, Wenger A, Suárez‐Castro AF, Gelfand S and Ahmadia GN (2021) A global map of human pressures on tropical coral reefs. Conservation Letters 15, e12858.

[r8] Ayala-Orozco B, Rosell JA, Merçon J, Bueno I, Alatorre-Frenk G, Langle-Flores A and Lobato A (2018) Challenges and strategies in place-based multi-stakeholder collaboration for sustainability: Learning from experiences in the Global South. Sustainability 10, 3217.

[r9] Bahr KD, Jokiel PL and Toonen RJ (2015) The unnatural history of Kāne‘ohe Bay: Coral reef resilience in the face of centuries of anthropogenic impacts. PeerJ 3, e950. 26020007 10.7717/peerj.950 PMC4435448

[r10] Bartley R, Corfield JP, Hawdon AA, Kinsey-Henderson AE, Abbott BN, Wilkinson SN and Keen RJ (2014) Can changes to pasture management reduce runoff and sediment loss to the Great Barrier Reef? The results of a 10-year study in the Burdekin catchment, Australia. Rangeland Journal 36, 67–84.

[r11] Bass D, Stentiford GD, Wang H-C, Koskella B and Tyler CR (2019) The pathobiome in animal and plant diseases. Trends in Ecology and Evolution 34, 996–1008. 31522755 10.1016/j.tree.2019.07.012 PMC7479508

[r12] Beer A, McKenzie F, Blažek J, Sotarauta M and Ayres S (2020) Every Place Matters: Towards Effective Place-Based Policy. Abingdon: Routledge.

[r13] Bellwood DR, Hughes TP, Folke C and Nyström M (2004) Confronting the coral reef crisis. Nature 429, 827–833. 15215854 10.1038/nature02691

[r14] Bessell-Browne P, Negri AP, Fisher R, Clode PL and Jones R (2017) Cumulative impacts: Thermally bleached corals have reduced capacity to clear deposited sediment. Scientific Reports 7, 2716. 28578383 10.1038/s41598-017-02810-0 PMC5457406

[r15] Birkeland CE, Green A, Fenner D, Squair C and Dahl AL (2013) Substratum stability and coral reef resilience: Insights from 90 years of disturbances on a reef in American Samoa. Micronesica 2013, 1–16.

[r16] Bosch AC, O’Neill B, Sigge GO, Kerwath SE and Hoffman LC (2016) Heavy metals in marine fish meat and consumer health: A review. Journal of the Science of Food and Agriculture 96, 32–48. 26238481 10.1002/jsfa.7360

[r17] Boucher J and Friot D (2017) Primary Microplastics in the Oceans: A Global Evaluation of Sources. Gland: International Union for Conservation of Nature and Natural Resources.

[r18] Boxall AB, Rudd MA, Brooks BW, Caldwell DJ, Choi K, Hickmann S, Innes E, Ostapyk K, Staveley JP, Verslycke T and Ankley GT (2012) Pharmaceuticals and personal care products in the environment: What are the big questions? Environmental Health Perspectives 120, 1221–1229. 22647657 10.1289/ehp.1104477 PMC3440110

[r19] Bremer LL, Auerbach DA, Goldstein JH, Vogl AL, Shemie D, Kroeger T, Nelson JL, Benítez SP, Calvache A, Guimarães J, Herron C, Higgins J, Klemz C, León J, Sebastián Lozano J, Moreno PH, Nuñez F, Veiga F and Tiepolo G (2016) One size does not fit all: Natural infrastructure investments within the Latin American Water Funds Partnership. Ecosystem Services 17, 217–236.

[r20] Brown CJ, Jupiter SD, Lin H-Y, Albert S, Klein C, Maina JM, Tulloch VJD, Wenger AS and Mumby PJ (2017) Habitat change mediates the response of coral reef fish populations to terrestrial run-off. Marine Ecology Progress Series 576, 55–68.

[r21] Bunch MJ, Parkes M, Zubrycki K, Venema H, Hallstrom L, Neudorffer C, Berbés-Blázquez M and Morrison K (2014) Watershed management and public health: An exploration of the intersection of two fields as reported in the literature from 2000 to 2010. Environmental Management 54, 240–254. 24938794 10.1007/s00267-014-0301-3

[r22] Burke L, Reytar K, Spalding M and Perry A (2011) Reefs at Risk Revisited. Washington, DC: World Resources Institute.

[r23] Cadham JC, Thomas RL, Khawlie M and Kawass I (2005) Environmental management of the waters of the El-Kabir River and the associated Akkar watershed. Lakes & Reservoirs: Research and Management 10, 141–146.

[r24] Cann KF, Thomas DR, Salmon RL, Wyn-Jones AP and Kay D (2013) Extreme water-related weather events and waterborne disease. Epidemiology and Infection 141, 671–686. 22877498 10.1017/S0950268812001653 PMC3594835

[r25] Carlson RR, Foo SA and Asner GP (2019) Land use impacts on coral reef health: A ridge-to-reef perspective. Frontiers in Marine Science 6, 562.

[r26] Cesar H, Burke L and Pet-Soede L (2003) The Economics of Worldwide Coral Reef Degradation. Zeist: Cesar Environmental Economics Consulting.

[r27] Chase C and Ngure FM (2016) *Multisectoral Approaches to Improving Nutrition: Water, Sanitation, and Hygiene.* Water and Sanitation Program Technical Paper 102935. Washington, DC: The World Bank. Available at https://documents1.worldbank.org/curated/en/881101468196156182/pdf/102935-WSP-Box394845B-PUBLIC-ADD-SERIES-Water-and-Sanitation-Program-WSP.pdf.

[r28] Claar DC, Starko S, Tietjen KL, Epstein HE, Cunning R, Cobb KM, Baker AC, Gates RD and Baum JK (2020) Dynamic symbioses reveal pathways to coral survival through prolonged heatwaves. Nature Communications 11, 6097. 10.1038/s41467-020-19169-y PMC7723047 33293528

[r29] Coral Reef Alliance (2020) Coral Reefs in Roatán Thrive with Clean Water. Available at https://coral.org/en/blog/coral-reefs-in-roatan-thrive-with-clean-water/ (accessed 30 March 2023).

[r30] Cox EF and Ward S (2002) Impact of elevated ammonium on reproduction in two Hawaiian scleractinian corals with different life history patterns. Marine Pollution Bulletin 44, 1230–1235. 12523521 10.1016/s0025-326x(02)00213-8

[r31] Crain CM, Halpern BS, Beck MW and Kappel CV (2009) Understanding and managing human threats to the coastal marine environment. Annals of the New York Academy of Sciences 1162, 39–62. 19432644 10.1111/j.1749-6632.2009.04496.x

[r32] Davidson SL and De Loë RC (2014) Watershed governance: Transcending boundaries. Water Alternatives 7, 367–387.

[r33] de Freitas LD, de Moraes JFL, da Costa AM, Martins LL, Silva BM, Avanzi JC and Uezu A (2022) How far can nature-based solutions increase water supply resilience to climate change in one of the most important Brazilian watersheds? Earth 3, 748–767.

[r34] Douglas I (1967) Man, vegetation and the sediment yields of rivers. Nature 215, 925–928.

[r35] Downs CA, Kramarsky-Winter E, Segal R, Fauth J, Knutson S, Bronstein O, Ciner FR, Jeger R, Lichtenfeld Y, Woodley CM, Pennington P, Cadenas K, Kushmaro A and Loya Y (2016) Toxicopathological effects of the sunscreen UV filter, oxybenzone (benzophenone-3), on coral planulae and cultured primary cells and its environmental contamination in Hawaii and the U.S. Virgin Islands. Archives of Environmental Contamination and Toxicology 70, 265–288. 26487337 10.1007/s00244-015-0227-7

[r36] Dudgeon D, Arthington AH, Gessner MO, Kawabata Z-I, Knowler DJ, Lévêque C, Naiman RJ, Prieur-Richard A-H, Soto D, Stiassny MLJ and Sullivan CA (2006) Freshwater biodiversity: Importance, threats, status and conservation challenges. Biological Reviews 81, 163–182. 16336747 10.1017/S1464793105006950

[r37] Edinger EN, Jompa J, Limmon GV, Widjatmoko W and Risk MJ (1998) Reef degradation and coral biodiversity in Indonesia: Effects of land-based pollution, destructive fishing practices and changes over time. Marine Pollution Bulletin 36, 617–630.

[r38] Edinger EN, Limmon GV, Jompa J, Widjatmoko W, Heikoop JM and Risk MJ (2000) Normal coral growth rates on dying reefs: Are coral growth rates good indicators of reef health? Marine Pollution Bulletin 40, 404–425.

[r39] Fabricius KE (2005) Effects of terrestrial runoff on the ecology of corals and coral reefs review and synthesis. Marine Pollution Bulletin 50, 125–146. 15737355 10.1016/j.marpolbul.2004.11.028

[r40] Fisher J (2008) Women in water supply, sanitation and hygiene programmes. Proceedings of the Institution of Civil Engineers – Municipal Engineer 161, 223–229.

[r41] Fisher R, Bessell-Browne P and Jones R (2019) Synergistic and antagonistic impacts of suspended sediments and thermal stress on corals. Nature Communications 10, 2346–2349. 10.1038/s41467-019-10288-9 PMC6538670 31138792

[r42] Fleming LE, Broad K, Clement A, Dewailly E, Elmir S, Knap A, Pomponi SA, Smith S, Solo Gabriele H and Walsh P (2006) Oceans and human health: Emerging public health risks in the marine environment: The oceans and human health. Marine Pollution Bulletin 53, 545–560. 16996542 10.1016/j.marpolbul.2006.08.012 PMC2573863

[r43] Freeman MC, Pringle CM and Jackson CR (2007) Hydrologic connectivity and the contribution of stream headwaters to ecological integrity at regional scales. Journal of the American Water Resources Association 43, 5–14.

[r44] Fuller R, Landrigan PJ, Balakrishnan K, Bathan G, Bose-O’Reilly S, Brauer M, Caravanos J, Chiles T, Cohen A, Corra L, Cropper M, Ferraro G, Hanna J, Hanrahan D, Hu H, Hunter D, Janata G, Kupka R, Lanphear B, Lichtveld M, Martin K, Mustapha A, Sanchez-Triana E, Sandilya K, Schaefli L, Shaw J, Seddon J, Suk W, Téllez-Rojo MM and Yan C (2022) Pollution and health: A progress update. Lancet Planetary Health 6, e535–e547. 35594895 10.1016/S2542-5196(22)00090-0 PMC11995256

[r45] Galloway TS (2015) Micro- and nano-plastics and human health. In Bergmann M, Gutow L and Klages M (eds), Marine Anthropogenic Litter. Cham: Springer Nature, pp. 343–366.

[r46] Garcia RN, Chung KW, DeLorenzo ME and Curran MC (2014) Individual and mixture effects of caffeine and sulfamethoxazole on the daggerblade grass shrimp *Palaemonetes pugio* following maternal exposure. Environmental Toxicology and Chemistry 33, 2120–2125. 24932500 10.1002/etc.2669

[r47] Gilmour J (1999) Experimental investigation into the effects of suspended sediment on fertilisation, larval survival and settlement in a scleractinian coral. Marine Biology 135, 451–462.

[r48] Golbuu Y, Fabricius K, Victor S and Richmond RH (2008) Gradients in coral reef communities exposed to muddy river discharge in Pohnpei, Micronesia. Estuarine, Coastal and Shelf Science 76, 14–20.

[r49] Golden CD, Allison EH, Cheung WWL, Dey MM, Halpern BS, McCauley DJ, Smith M, Vaitla B, Zeller D and Myers SS (2016) Nutrition: Fall in fish catch threatens human health. Nature 534, 317–320. 27306172 10.1038/534317a

[r50] Goldman-Benner RL, Benitez S, Boucher T, Calvache A, Daily G, Kareiva P, Kroeger T and Ramos A (2012) Water funds and payments for ecosystem services: Practice learns from theory and theory can learn from practice. Oryx 46, 55–63.

[r51] Gräslund S and Bengtsson BE (2001) Chemicals and biological products used in South-East Asian shrimp farming, and their potential impact on the environment—A review. Science of the Total Environment 280, 93–131. 11763276 10.1016/s0048-9697(01)00818-x

[r52] Harrison PL and Ward S (2001) Elevated levels of nitrogen and phosphorus reduce fertilisation success of gametes from scleractinian reef corals. Marine Biology 139, 1057–1068.

[r53] He Q and Silliman BR (2019) Climate change, human impacts, and coastal ecosystems in the anthropocene. Current Biology 29, R1021–R1035. 31593661 10.1016/j.cub.2019.08.042

[r54] Herrera D, Ellis A, Fisher B, Golden CD, Johnson K, Mulligan M, Pfaff A, Treuer T and Ricketts TH (2017) Upstream watershed condition predicts rural children’s health across 35 developing countries. Nature Communications 8, 811–818. 10.1038/s41467-017-00775-2 PMC5634511 28993648

[r55] Hess S, Wenger AS, Ainsworth TD and Rummer JL (2015) Exposure of clownfish larvae to suspended sediment levels found on the Great Barrier Reef: Impacts on gill structure and microbiome. Scientific Reports 5, 10561. 26094624 10.1038/srep10561 PMC5392994

[r56] Hicks CC, Cohen PJ, Graham NAJ, Nash KL, Allison EH, D’Lima C, Mills DJ, Roscher M, Thilsted SH, Thorne-Lyman AL and MacNeil MA (2019) Harnessing global fisheries to tackle micronutrient deficiencies. Nature 574, 95–98. 31554969 10.1038/s41586-019-1592-6

[r57] Hofstra N (2011) Quantifying the impact of climate change on enteric waterborne pathogen concentrations in surface water. Current Opinion in Environmental Sustainability 3, 471–479.

[r58] Horwitz P and Finlayson CM (2011) Wetlands as settings for human health: Incorporating ecosystem services and health impact assessment into water resource management. Bioscience 61, 678–688.

[r59] Huang W, Chen M, Song B, Deng J, Shen M, Chen Q, Zeng G and Liang J (2021) Microplastics in the coral reefs and their potential impacts on corals: A mini-review. Science of the Total Environment 762, 143112. 33172634 10.1016/j.scitotenv.2020.143112

[r60] Huitema D, Mostert E, Egas W, Moellenkamp S, Pahl-Wostl C and Yalcin R (2009) Adaptive water governance: Assessing the institutional prescriptions of adaptive (co-)management from a governance perspective and defining a research agenda. Ecology and Society 14, 26.

[r61] Jenkins A, Capon A, Negin J, Marais B, Sorrell T, Parkes M and Horwitz P (2018a) Watersheds in planetary health research and action. Lancet Planetary Health 2, e510–e511. 30526933 10.1016/S2542-5196(18)30228-6

[r62] Jenkins A, Horwitz P and Arabena K (2018b) My island home: Place-based integration of conservation and public health in Oceania. Environmental Conservation 45, 125–136.

[r63] Jenkins AP and Jupiter S (2015) Natural disasters, health and wetlands. In Finlayson CM, Horwitz P and Weinstein P (eds), Wetlands and Human Health. Dordrecht: Springer, pp. 169–191.

[r64] Jenkins AP, Jupiter S, Mueller U, Jenney A, Vosaki G, Rosa V, Naucukidi A, Mulholland K, Strugnell R, Kama M and Horwitz P (2016) Health at the sub-catchment scale: Typhoid and its environmental determinants in central division, Fiji. EcoHealth 13, 633–651. 27557784 10.1007/s10393-016-1152-6

[r65] Jenkins AP, Jupiter SD, Qauqau I and Atherton J (2010) Importance of ecosystem-based management for conserving aquatic migratory pathways on tropical high islands: A case study from Fiji. Aquatic Conservation: Marine and Freshwater Ecosystems 20, 224–238.

[r66] Jones R, Fisher R and Bessell-Browne P (2019) Sediment deposition and coral smothering. PLoS One 2019, e0216248. 10.1371/journal.pone.0216248 PMC6584000 31216275

[r67] Jordan SJ and Benson WH (2020) Sustainable watersheds: Integrating ecosystem services and public health. Environmental Health Insights 9, EHI-S19586. 10.4137/EHI.S19586 PMC4425197 25987844

[r68] Jupiter SD, Jenkins AP, Lee Long WJ, Maxwell SL, Carruthers TJB, Hodge KB, Govan H, Tamelander J and Watson JEM (2014) Principles for integrated island management in the tropical Pacific. Pacific Conservation Biology 20, 193–205.

[r69] Jupiter SD, Wenger A, Klein CJ, Albert S, Mangubhai S, Nelson J, Teneva L, Tulloch VJ, White AT and Watson JEM (2017) Opportunities and constraints for implementing integrated land–sea management on islands. Environmental Conservation 44, 254–266.

[r70] Kauffman CM (2014) Financing watershed conservation: Lessons from Ecuador’s evolving water trust funds. Agricultural Water Management 145, 39–49.

[r71] Kawarazuka N and Béné C (2010) Linking small-scale fisheries and aquaculture to household nutritional security: An overview. Food Security 2, 343–357.

[r72] Kirstein IV, Kirmizi S, Wichels A, Garin-Fernandez A, Erler R, Löder M and Gerdts G (2016) Dangerous hitchhikers? Evidence for potentially pathogenic *Vibrio* spp. on microplastic particles. Marine Environmental Research 120, 1–8. 27411093 10.1016/j.marenvres.2016.07.004

[r73] Kondolf GM (1994) Geomorphic and environmental effects of instream gravel mining. Landscape and Urban Planning 28, 225–243.

[r74] Koop K, Booth D, Broadbent A, Brodie J, Bucher D, Capone D, Coll J, Dennison W, Erdmann M, Harrison P, Hoegh-Guldberg O, Hutchings P, Jones GB, Larkum AWD, O’Neil J, Steven A, Tentori E, Ward S, Williamson J and Yellowlees D (2001) ENCORE: The effect of nutrient enrichment on coral reefs. Synthesis of results and conclusions. Marine Pollution Bulletin 42, 91–120. 11381890 10.1016/s0025-326x(00)00181-8

[r75] Kovacs SD, Mullholland K, Bosch J, Campbell H, Forouzanfar MH, Khalil I, Lim S, Liu L, Maley SN, Mathers CD, Matheson A, Mokdad AH, O’Brien K, Parashar U, Schaafsma TT, Steele D, Hawes SE and Grove JT (2015) Deconstructing the differences: A comparison of GBD 2010 and CHERG’s approach to estimating the mortality burden of diarrhea, pneumonia, and their etiologies. BMC Infectious Diseases 15, 16–16. 25592774 10.1186/s12879-014-0728-4 PMC4305232

[r76] Kroon FJ, Berry KL, Brinkman DL, Kookana R, Leusch FD, Melvin SD, Neale PA, Negri AP, Puotinen M, Tsang JJ and van de Merwe JP (2020) Sources, presence and potential effects of contaminants of emerging concern in the marine environments of the Great Barrier Reef and Torres Strait, Australia. Science of the Total Environment 719, 135140. 31859059 10.1016/j.scitotenv.2019.135140

[r77] Kroon FJ, Schaffelke B and Bartley R (2014) Informing policy to protect coastal coral reefs: Insight from a global review of reducing agricultural pollution to coastal ecosystems. Marine Pollution Bulletin 85, 33–41. 24975091 10.1016/j.marpolbul.2014.06.003

[r78] Lamb JB, Van De Water JAJM, Bourne DG, Altier C, Hein MY, Fiorenza EA, Abu N, Jompa J and Harvell CD (2017) Seagrass ecosystems reduce exposure to bacterial pathogens of humans, fishes, and invertebrates. Science 355, 731–733. 28209895 10.1126/science.aal1956

[r79] Lamb JB, Willis BL, Fiorenza EA, Couch CS, Howard R, Rader DN, True JD, Kelly LA, Ahmad A, Jompa J and Harvell CD (2018) Plastic waste associated with disease on coral reefs. Science 359, 460–462. 29371469 10.1126/science.aar3320

[r80] Landrigan PJ, Stegeman JJ, Fleming LE, Allemand D, Anderson DM, Backer LC, Brucker-Davis F, Chevalier N, Corra L, Czerucka D, Bottein M-YD, Demeneix B, Depledge M, Deheyn DD, Dorman CJ, Fénichel P, Fisher S, Gaill F, Galgani F, Gaze WH, Giuliano L, Grandjean P, Hahn ME, Hamdoun A, Hess P, Judson B, Laborde A, McGlade J, Mu J, Mustapha A, Neira M, Noble RT, Pedrotti ML, Reddy C, Rocklöv J, Scharler UM, Shanmugam H, Taghian G, Van De Water JAJM, Vezzulli L, Weihe P, Zeka A, Raps H and Rampal P (2020) Human health and ocean pollution. Annals of Global Health 86, 1–64. 33354517 10.5334/aogh.2831 PMC7731724

[r81] Lane MB (2008) Strategic coastal governance issues in Fiji: The challenges of integration. Marine Policy 32, 856–866.

[r82] Lapointe BE, Thacker K, Hanson C and Getten L (2011) Sewage pollution in Negril, Jamaica: Effects on nutrition and ecology of coral reef macroalgae. Chinese Journal of Oceanology and Limnology 29, 775–789.

[r83] Lau CL, Smythe LD, Craig SB and Weinstein P (2010) Climate change, flooding, urbanisation and leptospirosis: Fuelling the fire? Transactions of the Royal Society of Tropical Medicine and Hygiene 104, 631–638. 20813388 10.1016/j.trstmh.2010.07.002

[r84] Le Grand HM and Fabricius KE (2011) Relationship of internal macrobioeroder densities in living massive Porites to turbidity and chlorophyll on the Australian Great Barrier Reef. Coral Reefs 30, 97–107.

[r85] Leder K, Openshaw JJ, Allotey P, Ansariadi A, Barker SF, Burge K, Clasen TF, Chown SL, Duffy GA, Faber PA, Fleming G, Forbes AB, French M, Greening C, Henry R, Higginson E, Johnston DW, Lappan R, Lin A, Luby SP, McCarthy D, O’Toole JE, Ramirez-Lovering D, Reidpath DD, Simpson JA, Sinharoy SS, Sweeney R, Taruc RR, Tela A, Turagabeci AR, Wardani J, Wong T and Brown R (2021) Study design, rationale and methods of the Revitalising Informal Settlements and their Environments (RISE) study: A cluster randomised controlled trial to evaluate environmental and human health impacts of a water-sensitive intervention in informal settlements in Indonesia and Fiji. BMJ Open 11, e042850. 10.1136/bmjopen-2020-042850 PMC7798802 33419917

[r86] Levy K, Smith SM and Carlton EJ (2018) Climate change impacts on waterborne diseases: Moving toward designing interventions. Current Environmental Health Reports 5, 272–282. 29721700 10.1007/s40572-018-0199-7 PMC6119235

[r87] Li X, Tian Y, Xu C and Cheng B (2019) The impact of marine pollution control on the output value of marine fisheries based on the spatial econometric model. Journal of Coastal Research 98, 381–384.

[r88] Liao H, Yen JY, Guan Y, Ke D and Liu C (2020) Differential responses of stream water and bed sediment microbial communities to watershed degradation. Environment International 134, 105198. 31704564 10.1016/j.envint.2019.105198

[r89] Littman RA, Fiorenza EA, Wenger AS, Berry KLE, van de Water JAJM, Nguyen L, Aung ST, Parker DM, Rader DN, Harvell CD and Lamb JB (2020) Coastal urbanization influences human pathogens and microdebris contamination in seafood. Science of the Total Environment 736, 139081. 32504866 10.1016/j.scitotenv.2020.139081

[r90] Liu Y, Engel BA, Flanagan DC, Gitau MW, McMillan SK and Chaubey I (2017) A review on effectiveness of best management practices in improving hydrology and water quality: Needs and opportunities. Science of the Total Environment 601–602, 580–593. 28575835 10.1016/j.scitotenv.2017.05.212

[r91] Lotze HK, Lenihan HS, Bourque BJ, Bradbury RH, Cooke RG, Kay MC, Kidwell SM, Kirby MX, Peterson CH and Jackson JBC (2006) Depletion, degradation, and recovery potential of estuaries and coastal seas. Science 312, 1806–1809. 16794081 10.1126/science.1128035

[r92] Loya Y (2004) The coral reefs of Eliat-past, present and future: Three decades of coral community structure studies. In Rosenberg E and Loya Y (eds), Coral Reef Health and Disease. New York: Springer, pp. 1–34.

[r93] Lu Y, Yuan J, Lu X, Su C, Zhang Y, Wang C, Cao X, Li Q, Su J, Ittekkot V, Garbutt RA, Bush S, Fletcher S, Wagey T, Kachur A and Sweijd N (2018) Major threats of pollution and climate change to global coastal ecosystems and enhanced management for sustainability. Environmental Pollution 239, 670–680. 29709838 10.1016/j.envpol.2018.04.016

[r94] Lusher A, Hollman P and Mendoza-Hill J (2017) Microplastics in Fisheries and Aquaculture: Status of Knowledge on Their Occurrence and Implications for Aquatic Organisms and Food Safety. FAO fisheries and aquaculture technical paper. Rome: Food and Agriculture Organisation of the United Nations.

[r95] MacLeod M, Arp HPH, Tekman MB and Jahnke A (2021) The global threat from plastic pollution. Science 373, 61–65. 34210878 10.1126/science.abg5433

[r96] Malan HL, Appleton CC, Day JA and Dini J (2009) Wetlands and invertebrate disease hosts: Are we asking for trouble? Water SA 35, 753–768.

[r97] Mangubhai S, Sykes H, Lovell E, Brodie G, Jupiter S, Morris C, Lee S, Loganimoce E, Rashni B, Lal R, Nand Y and Qauqau I (2018) Fiji: Coastal and marine ecosystems. In Sheppard C (ed.), World Seas: An Environmental Evaluation Volume II: The Indian Ocean to the Pacific. London: Elsevier, pp. 765–792.

[r98] Maranho LA, Moreira LB, Baena-Nogueras RM, Lara-Martín PA, DelValls TA and Martín-Díaz ML (2014) A candidate short-term toxicity test using *Ampelisca brevicornis* to assess sublethal responses to pharmaceuticals bound to marine sediments. Archives of Environmental Contamination and Toxicology 68, 237–258. 25227176 10.1007/s00244-014-0080-0

[r99] McDowell RW and Wilcock RJ (2008) Water quality and the effects of different pastoral animals. New Zealand Veterinary Journal 56, 289–296. 19043466 10.1080/00480169.2008.36849

[r100] McFarlane RA, Horwitz P, Arabena K, Capon A, Jenkins A, Jupiter S, Negin J, Parkes MW and Saketa S (2019) Ecosystem services for human health in Oceania. Ecosystem Services 39, 100976.

[r101] McField M, Kramer P, Giró Petersen A, Soto M, Drysdale I, Craig N and Rueda-Flores M (2020) 2020 Mesoamerican Reef Report Card. Healthy Reefs Initiative. Available at www.healthyreefs.org (accessed 09 January 2022).

[r102] McField M, Soto M, Craig N, Giró A, Drysdale I, Rueda-Flores M, Castillo M, Kramer P and Roth L (2022) 2022 Essentail Report Card for the Mesoamerican Reef. Healthy Reefs Initiative. Available at www.healthyreefs.org (accessed 15 September 2022).

[r103] McGrane SJ (2016) Impacts of urbanisation on hydrological and water quality dynamics, and urban water management: A review. Hydrological Sciences Journal 61, 2295–2311.

[r104] McKenzie LJ and Yoshida RL (2020) Over a decade monitoring Fiji’s seagrass condition demonstrates resilience to anthropogenic pressures and extreme climate events. Marine Pollution Bulletin 160, 111636. 33181923 10.1016/j.marpolbul.2020.111636

[r105] McManus JW, Meñez LAB, Kesner-Reyes KN, Vergara SG and Ablan MC (2000) Coral reef fishing and coral-algal phase shifts: Implications for global reef status. ICES Journal of Marine Science 57, 572–578.

[r106] Meals DW, Dressing SA and Davenport TE (2010) Lag time in water quality response to best management practices: A review. Journal of Environmental Quality 39, 85–96. 20048296 10.2134/jeq2009.0108

[r193] Mitchell JR, Mitchell RK, Hunt RA, Townsend DM and Lee JH (2022) Stakeholder engagement, knowledge problems and ethical challenges. Journal of Business Ethics 175, 75–94.

[r107] Moberg F and Folke C (1999) Ecological goods and services of coral reef ecosystems. Ecological Economics 29, 215–233.

[r108] Morrison TH (2017) Evolving polycentric governance of the Great Barrier Reef. Proceedings of the National Academy of Sciences 114, E3013–E3021. 10.1073/pnas.1620830114 PMC5393255 28348238

[r109] Moustaka M, Langlois TJ, McLean D, Bond T, Fisher R, Fearns P, Dorji P and Evans RD (2018) The effects of suspended sediment on coral reef fish assemblages and feeding guilds of north-west Australia. Coral Reefs 37, 659–673.

[r110] Muchapondwa E, Stage J, Mungatana E and Kumar P (2018) Lessons from applying market-based incentives in watershed management. Water Economics and Policy 4, 1850011.

[r111] Müller A, Österlund H, Marsalek J and Viklander M (2020) The pollution conveyed by urban runoff: A review of sources. Science of the Total Environment 709, 136125. 31905584 10.1016/j.scitotenv.2019.136125

[r112] Mumby PJ, Hastings A and Edwards HJ (2007) Thresholds and the resilience of Caribbean coral reefs. Nature 450, 98–101. 17972885 10.1038/nature06252

[r113] Nalley EM, Tuttle LJ, Barkman AL, Conklin EE, Wulstein DM, Richmond RH and Donahue MJ (2021) Water quality thresholds for coastal contaminant impacts on corals: A systematic review and meta-analysis. Science of the Total Environment 794, 148632. 34323749 10.1016/j.scitotenv.2021.148632

[r114] Negri AP, Smith LD, Webster NS and Heyward AJ (2002) Understanding ship-grounding impacts on a coral reef: Potential effects of anti-foulant paint contamination on coral recruitment. Marine Pollution Bulletin 44, 111–117. 11981977 10.1016/s0025-326x(01)00128-x

[r115] Nelson S, Jenkins A, Jupiter SD, Horwitz P, Mangubhai S, Abimbola S, Ratu A, Naivalulevu T and Negin J (2022) Predicting climate-sensitive water-related disease trends based on health, seasonality and weather data in Fiji. Journal of Climate Change and Health 6, 100112.

[r116] O’Neill J (2016) Review on Antimicrobial Resistance: Tackling Drug-Resistant Infections Globally: Final Report and Recommendations. Government of the United Kingdom. Available at https://amr-review.org/sites/default/files/160525_Final%20paper_with%20cover.pdf (accessed 09 January 2022).

[r117] Olmstead S and Zheng J (2021) Water pollution control in developing countries: Policy instruments and empirical evidence. Review of Environmental Economics and Policy 15, 261–280.

[r118] Olsen S and Christie P (2000) What are we learning from tropical coastal management experiences? Coastal Management 28, 5–18.

[r119] Orth RJ, Carruthers TJB, Dennison WC, Duarte CM, Fourqurean JW, Heck KL, Hughes AR, Kendrick GA, Kenworthy WJ, Olyarnik S, Short FT, Waycott M and Williams SL (2006) A global crisis for seagrass ecosystems. Bioscience 56, 987–996.

[r194] Oteros-Rozas E, Martin-Lopez B, Daw TM, Bohensky EL, Butler JR, Hill R, Martin-Ortega J, Quinlan A, Ravera F, Ruiz-Mallen I and Thyresson M (2015) Participatory scenario planning in place-based social-ecological research: insights and experiences from 23 case studies. Ecology and Society 20, 32.

[r120] Parkes MW and Horwitz P (2009) Water, ecology and health: Ecosystems as settings for promoting health and sustainability. Health Promotion International 24, 94–102. 19171669 10.1093/heapro/dan044

[r121] Parkes MW, Morrison KE, Bunch MJ, Hallström LK, Neudoerffer RC, Venema HD and Waltner-Toews D (2010) Towards integrated governance for water, health and social–ecological systems: The watershed governance prism. Global Environmental Change 20, 693–704.

[r122] Peng M and Oleson KLL (2017) Beach recreationalists’ willingness to pay and economic implications of coastal water quality problems in Hawaii. Ecological Economics 136, 41–52.

[r123] Peters NE and Meybeck M (2000) Water quality degradation effects on freshwater availability: Impacts of human activities. Water International 25, 185–193.

[r124] Philipp E and Fabricius K (2003) Photophysiological stress in scleractinian corals in response to short-term sedimentation. Journal of Experimental Marine Biology and Ecology 287, 57–78.

[r125] Price JI and Heberling MT (2018) The effects of source water quality on drinking water treatment costs: A review and synthesis of empirical literature. Ecological Economics 151, 195–209. 30008516 10.1016/j.ecolecon.2018.04.014 PMC6040680

[r126] Primavera JH (2006) Overcoming the impacts of aquaculture on the coastal zone. Ocean and Coastal Management 49, 531–545.

[r127] Prüss-Ustün A, Wolf J, Bartram J, Clasen T, Cumming O, Freeman MC, Gordon B, Hunter PR, Medlicott K and Johnston R (2019) Burden of disease from inadequate water, sanitation and hygiene for selected adverse health outcomes: An updated analysis with a focus on low- and middle-income countries. International Journal of Hygiene and Environmental Health 222, 765–777. 31088724 10.1016/j.ijheh.2019.05.004 PMC6593152

[r128] Rabalais NN, Turner RE, Díaz RJ and Justić D (2009) Global change and eutrophication of coastal waters. ICES Journal of Marine Science 66, 1528–1537.

[r129] Ragosta G, Evensen C, Atwill ER, Walker M, Ticktin T, Asquith A and Tate KW (2011) Risk factors for elevated Enterococcus concentrations in a rural tropical island watershed. Journal of Environmental Management 92, 1910–1915. 21530065 10.1016/j.jenvman.2011.02.017

[r130] Ranjbar Jafarabadi A, Riyahi Bakhtiari A, Aliabadian M, Laetitia H, Shadmehri Toosi A and Yap CK (2018) First report of bioaccumulation and bioconcentration of aliphatic hydrocarbons (AHs) and persistent organic pollutants (PAHs, PCBs and PCNs) and their effects on alcyonacea and scleractinian corals and their endosymbiotic algae from the Persian Gulf, Iran: Inter and intra-species differences. Science of the Total Environment 627, 141–157. 29426136 10.1016/j.scitotenv.2018.01.185

[r131] Redding JE, Myers-Miller RL, Baker DM, Fogel M, Raymundo LJ and Kim K (2013) Link between sewage-derived nitrogen pollution and coral disease severity in Guam. Marine Pollution Bulletin 73, 57–63. 23816306 10.1016/j.marpolbul.2013.06.002

[r132] Reef Resilience Network (2021) Honduras – Wastewater Pollution: Sanitation Best Management Practices in West End, Honduras Reef Resilience Network. Available at https://reefresilience.org/case-studies/honduras-wastewater-pollution/ (accessed 09 January 2022).

[r133] Rehman K, Fatima F, Waheed I and Akash MSH (2018) Prevalence of exposure of heavy metals and their impact on health consequences. Journal of Cellular Biochemistry 119, 157–184. 28643849 10.1002/jcb.26234

[r134] Ricardo GF, Jones RJ, Clode PL, Humanes A, Giofre N and Negri AP (2018) Sediment characteristics influence the fertilisation success of the corals *Acropora tenuis* and *Acropora millepora*. Marine Pollution Bulletin 135, 941–953. 30301119 10.1016/j.marpolbul.2018.08.001

[r135] Rice MM, Maher RL, Correa AMS, Moeller HV, Lemoine NP, Shantz AA, Burkepile DE and Silbiger NJ (2020) Macroborer presence on corals increases with nutrient input and promotes parrotfish bioerosion. Coral Reefs 39, 409–418.

[r136] Richmond RH, Golbuu Y and Shelton AJ (2019) Successful management of coral reef-watershed network. In Wolanski E, Day JW, Elliot M and Ramachandran R (eds), Coasts and Estuaries: The Future. Amsterdam: Elsevier, pp. 445–459.

[r137] Rogers CS (1990) Responses of coral reefs and reef organisms to sedimentation. Marine Ecology Progress Series 62, 185–202.

[r138] Sahavacharin A, Sompongchaiyakul P and Thaitakoo D (2022) The effects of land-based change on coastal ecosystems. Landscape and Ecological Engineering 18, 351–366.

[r139] Sale PF, Agardy T, Ainsworth CH, Feist BE, Bell JD, Christie P, Hoegh-Guldberg O, Mumby PJ, Feary DA, Saunders MI, Daw TM, Foale SJ, Levin PS, Lindeman KC, Lorenzen K, Pomeroy RS, Allison EH, Bradbury RH, Corrin J, Edwards AJ, Obura DO, Sadovy de Mitcheson YJ, Samoilys MA and Sheppard CRC (2014) Transforming management of tropical coastal seas to cope with challenges of the 21st century. Marine Pollution Bulletin 85, 8–23. 24997002 10.1016/j.marpolbul.2014.06.005

[r140] Semenza JC (2020) Cascading risks of waterborne diseases from climate change. Nature Immunology 21, 484–487. 32313241 10.1038/s41590-020-0631-7

[r141] Shore-Maggio A, Aeby GS and Callahan SM (2018) Influence of salinity and sedimentation on Vibrio infection of the Hawaiian coral *Montipora capitata*. Diseases of Aquatic Organisms 128, 63–71. 29565254 10.3354/dao03213

[r142] Shortle JS and Horan RD (2001) The economics of nonpoint pollution control. Journal of Economic Surveys 15, 255–289.

[r143] Shumway NR (2020) Impact Mitigation in Marine and Coastal Environments: Policy Challenges and Shortfalls. PhD thesis, School of Earth and Environmental Sciences. The University of Queensland, Brisbane.

[r144] Shuval H (2003) Estimating the global burden of thalassogenic diseases: Human infectious diseases caused by wastewater pollution of the marine environment. Journal of Water and Health 1, 53–64. 15382734

[r145] Sindermann CJ (2006) Coastal Pollution Effects on Living Resources and Humans. Boca Raton: CRC Press/Taylor & Francis.

[r146] Singh RBK, Hales S, De Wet N, Raj R, Hearnden M and Weinstein P (2001) The influence of climate variation and change on diarrheal disease in the Pacific Islands. Environmental Health Perspectives 109, 155–159. 11266326 10.1289/ehp.01109155 PMC1240636

[r147] Smith M, Love DC, Rochman CM and Neff RA (2018) Microplastics in seafood and the implications for human health. Current Environmental Health Reports 5, 375–386. 30116998 10.1007/s40572-018-0206-z PMC6132564

[r148] Sorenson SB, Morssink C and Campos PA (2011) Safe access to safe water in low income countries: Water fetching in current times. Social Science & Medicine 72, 1522–1526. 21481508 10.1016/j.socscimed.2011.03.010

[r149] Suárez‐Castro AF, Beyer HL, Kuempel CD, Linke S, Borrelli P and Hoegh‐Guldberg O (2021) Global forest restoration opportunities to foster coral reef conservation. Global Change Biology 27, 5238–5252. 34350684 10.1111/gcb.15811

[r150] Sundarambal P, Tkalich P and Balasubramanian R (2010) Impact of biomass burning on ocean water quality in Southeast Asia through atmospheric deposition: Eutrophication modeling. Atmospheric Chemistry and Physics 10, 11337–11357.

[r151] Sutherland KP, Shaban S, Joyner JL, Porter JW and Lipp EK (2011) Human pathogen shown to cause disease in the threatened eklhorn coral *Acropora palmata*. PLoS One 6, e23468. 21858132 10.1371/journal.pone.0023468 PMC3157384

[r152] Tarrant AM, Atkinson MJ and Atkinson S (2004) Effects of steroidal estrogens on coral growth and reproduction. Marine Ecology Progress Series 269, 121–129.

[r153] Taylor C, Pollard S, Rocks S and Angus A (2012) Selecting policy instruments for better environmental regulation: A critique and future research agenda. Environmental Policy and Governance 22, 268–292.

[r154] Tebbett SB and Bellwood DR (2019) Algal turf sediments on coral reefs what’s known and what’s next. Marine Pollution Bulletin 149, 110542. 31542595 10.1016/j.marpolbul.2019.110542

[r155] Teh LSL, Teh LCL and Sumaila UR (2013) A global estimate of the number of coral reef fishers. PLoS One 8, e65397. 23840327 10.1371/journal.pone.0065397 PMC3686796

[r156] Tengö M, Brondizio ES, Elmqvist T, Malmer P and Spierenburg M (2014) Connecting diverse knowledge systems for enhanced ecosystem governance: The multiple evidence base approach. Ambio 43, 579–591. 24659474 10.1007/s13280-014-0501-3 PMC4132468

[r157] The Nature Conservancy (TNC) and Goldman RL (2009) Ecosystem services and water funds: Conservation approaches that benefit people and biodiversity. Journal American Water Works Association 101, 20–22.

[r158] Thorburn PJ, Wilkinson SN and Silburn DM (2013) Water quality in agricultural lands draining to the Great Barrier Reef: A review of causes, management and priorities. Agriculture, Ecosystems & Environment 180, 4–20.

[r159] Todd PA, Ong X and Chou LM (2010) Impacts of pollution on marine life in Southeast Asia. Biodiversity and Conservation 19, 1063–1082.

[r160] Tuholske C, Halpern BS, Blasco G, Villasenor JC, Frazier M and Caylor K (2021) Mapping global inputs and impacts from of human sewage in coastal ecosystems. PLoS One 16, e0258898. 34758036 10.1371/journal.pone.0258898 PMC8580218

[r161] Turner NR and Renegar DA (2017) Petroleum hydrocarbon toxicity to corals: A review. Marine Pollution Bulletin 119, 1–16. 28502453 10.1016/j.marpolbul.2017.04.050

[r162] Turschwell MP, Connolly RM, Dunic JC, Sievers M, Buelow CA, Pearson RM, Tulloch VJD, Côté IM, Unsworth RKF, Collier CJ and Brown CJ (2021) Anthropogenic pressures and life history predict trajectories of seagrass meadow extent at a global scale. Proceedings of the National Academy of Sciences 118, e2110802118. 10.1073/pnas.2110802118 PMC8609331 34725160

[r163] United Nations Environment Programme (2012) Convention for the Protection and Development of the Marine Environment of the Wider Caribbean Region and its Protocols: Protocol Concerning Co-operation in Combating; Protocol Concerning Specially Protected Areas and Wildlife Oil Spills in the Wider Caribbean Region; Protocol Concerning Pollution from Land-Based Sources and Activities. Kingston: Regional Coordinating Unit of the United Nations Environment Programme - Caribbean Environment Programme. Available at https://wedocs.unep.org/20.500.11822/27875 (accessed 15 September 2022).

[r164] United Nations Inter-Agency Group for Child Mortality Estimation (UN-IGME) (2019) Levels and Trends in Child Mortality. New York: United Nations Children’s Fund. Available at https://www.unicef.org/media/60561/file/UN-IGME-child-mortality-report-2019.pdf (accessed 06 March 2022).

[r165] van Dam JW, Negri AP, Uthicke S and Mueller JF (2011) Chemical pollution on coral reefs: Exposure and ecological effects. In Sánchez-Bayo F, van den Brink PJ and Mann RM (eds), Ecological Impacts of Toxic Chemicals. Sharjah: Bentham Science Publishers, pp. 187–211.

[r166] van der Meij SET, Suharsono and Hoeksema BW (2010) Long-term changes in coral assemblages under natural and anthropogenic stress in Jakarta Bay (1920–2005). Marine Pollution Bulletin 60, 1442–1454. 20615515 10.1016/j.marpolbul.2010.05.011

[r167] Van Woesik R and Done TJ (1997) Coral communities and reef growth in the southern Great Barrier Reef. Coral Reefs 16, 103–115.

[r168] Vargas-Ángel B and Huntington B (2020) Status and Trends Assessment for Land-Based Sources of Pollution Impacts on Benthic Reef Communities in Faga‘alu Bay, American Samoa. National Oceanic and Atmospheric Administration Technical Memorandum NOAA-TM-NMFS-PIFSC-109. Honolulu: United States Department of Commerce. Available at https://repository.library.noaa.gov/view/noaa/27088 (accessed 15 December 2021).

[r169] Vega-Thurber R, Mydlarz LD, Brandt M, Harvell D, Weil E, Raymundo L, Willis BL, Langevin S, Tracy AM and Littman R (2020) Deciphering coral disease dynamics: Integrating host, microbiome, and the changing environment. Frontiers in Ecology and Evolution 8, 575927.

[r170] Vigerstol K, Abell R, Brauman K, Buytaert W and Vogl A (2021) Addressing water security through nature-based solutions. In Cassin J, Matthews JH and Gunn EL (eds), Nature-Based Solutions and Water Security. Amsterdam: Elsevier, pp. 37–62.

[r171] Wang G, Mang S, Cai H, Liu S, Zhang Z, Wang L and Innes JL (2016) Integrated watershed management: Evolution, development and emerging trends. Journal of Forestry Research 27, 967–994.

[r172] Wang J, Beusen AHW, Liu X and Bouwman AF (2020) Aquaculture production is a large, spatially concentrated source of nutrients in Chinese freshwater and coastal seas. Environmental Science and Technology 54, 1464–1474. 31642664 10.1021/acs.est.9b03340

[r173] Watkins YSD and Sallach JB (2021) Investigating the exposure and impact of chemical UV filters on coral reef ecosystems: Review and research gap prioritization. Integrated Environmental Assessment and Management 17, 967–981. 33734562 10.1002/ieam.4411

[r174] Wear SL (2016) Missing the boat: Critical threats to coral reefs are neglected at global scale. Marine Policy 74, 153–157.

[r175] Wear SL, Acuña V, McDonald R and Font C (2021) Sewage pollution, declining ecosystem health, and cross-sector collaboration. Biological Conservation 255, 109010.

[r176] Wear SL and Thurber RV (2015) Sewage pollution: Mitigation is key for coral reef stewardship. Annals of the New York Academy of Sciences 1355, 15–30. 25959987 10.1111/nyas.12785 PMC4690507

[r177] Weber M, de Beer D, Lott C, Polerecky L, Kohls K, Abed RMM, Ferdelman TG and Fabricius KE (2012) Mechanisms of damage to corals exposed to sedimentation. Proceedings of the National Academy of Sciences 109, E1558–E1567. 10.1073/pnas.1100715109 PMC3386076 22615403

[r178] Weber R, Watson A, Forter M and Oliaei F (2011) Persistent organic pollutants and landfills-a review of past experiences and future challenges. Waste Management & Research 29, 107–121. 21224404 10.1177/0734242X10390730

[r179] Wenger AS, Fabricius KE, Jones GP and Je B (2015) Effects of sedimentation, eutrophication, and chemical pollution on coral reef fishes. In Mora C (ed.), Ecology of Fishes on Coral Reefs. Cambridge: Cambridge University Press, pp. 145–153.

[r180] Wenger AS, Harris D, Weber S, Vaghi F, Nand Y, Naisilisili W, Hughes A, Delevaux J, Klein CJ, Watson J, Mumby PJ, Jupiter SD and Dhanjal‐Adams K (2020) Best‐practice forestry management delivers diminishing returns for coral reefs with increased land‐clearing. Journal of Applied Ecology 57, 2381–2392.

[r181] Wesseling I, Uychiaoco AJ, Aliño PM and Vermaat JE (2001) Partial mortality in porites corals: Variation among Philippine Reefs. International Review of Hydrobiology 86, 77–85.

[r182] West K and van Woesik R (2001) Spatial and temporal variance of river discharge on Okinawa (Japan) inferring the temporal impact on adjacent coral reefs. Marine Pollution Bulletin 42, 864–872. 11693640 10.1016/s0025-326x(01)00040-6

[r183] Woolhouse M, Waugh C, Perry MR and Nair H (2016) Global disease burden due to antibiotic resistance - State of the evidence. Journal of Global Health 6, 010306. 27350872 10.7189/jogh.06.010306 PMC4920009

[r184] World Health Organization (WHO) (2012) Global Costs and Benefits of Drinking-Water Supply and Sanitation Interventions to Reach the MDG Target and Universal Coverage. Geneva: World Health Organization. Available at https://apps.who.int/iris/bitstream/handle/10665/75140/WHO_HSE_WSH_12.01_eng.pdf (accessed 15 September 2022).

[r185] World Health Organization (WHO) (2014) Antimicrobial Resistance: An Emerging Water, Sanitation and Hygiene Issue. Geneva: World Health Organization. Available at https://www.who.int/publications/i/item/briefing-note-antimicrobial-reistance-an-emerging-water-sanitation-and-hygiene-issue (accessed 04 March 2022).

[r186] World Health Organization (WHO) (2015) WHO Estimates of the Global Burden of Foodborne Diseases: Foodborne Disease Burden Epidemiology Reference Group 2007–2015. Geneva: World Health Organization. Available at https://apps.who.int/iris/handle/10665/199350 (accessed 18 January 2021).

[r187] World Health Organization (WHO) (2016) Protecting Surface Water for Health: Identifying, Assessing and Managing Drinking-Water Quality Risks in Surface-Water Catchments. Geneva: World Health Organization. Available at https://www.who.int/publications/i/item/9789241510554 (accessed 18 January 2021).

[r188] World Health Organization (WHO) (2019) Safer Water, Better Health. Geneva: World Health Organization. Available at https://www.who.int/publications/i/item/9789241516891 (accessed 15 September 2022).

[r189] World Health Organization (WHO) and United Nations Children’s Fund (UNICEF) (2021) Progress on Household Drinking Water, Sanitation and Hygiene 2000–2020: Five Years into the SDGs. Geneva: World Health Organization and the United Nations Children’s Fund. Available at https://washdata.org/sites/default/files/2022-01/jmp-2021-wash-households_3.pdf (accessed 24 January 2022).

[r190] Wu NC and Seebacher F (2020) Effect of the plastic pollutant bisphenol A on the biology of aquatic organisms: A meta-analysis. Global Change Biology 26, 3821–3833. 32436328 10.1111/gcb.15127

[r191] Zettler ER, Mincer TJ and Amaral-Zettler LA (2013) Life in the “Plastisphere”: Microbial communities on plastic marine debris. Environmental Science and Technology 47, 7137–7146. 23745679 10.1021/es401288x

[r192] Zhang E, Kim M, Rueda L, Rochman C, VanWormer E, Moore J and Shapiro K (2022) Association of zoonotic protozoan parasites with microplastics in seawater and implications for human and wildlife health. Scientific Reports 12, 6532. 35474071 10.1038/s41598-022-10485-5 PMC9042925

